# Topically-applied collagen-binding serum albumin-fused interleukin-4 modulates wound microenvironment in non-healing wounds

**DOI:** 10.1038/s41536-023-00326-y

**Published:** 2023-09-11

**Authors:** Abigail L. Lauterbach, Rachel P. Wallace, Aaron T. Alpar, Kirsten C. Refvik, Joseph W. Reda, Ako Ishihara, Taryn N. Beckman, Anna J. Slezak, Yukari Mizukami, Aslan Mansurov, Suzana Gomes, Jun Ishihara, Jeffrey A. Hubbell

**Affiliations:** 1 Pritzker School of Molecular Engineering, University of Chicago, Chicago, IL 60637 USA; 2 Department of Bioengineering, Imperial College London, London, W12 0BZ UK; 3 Committee on Molecular Metabolism and Nutrition, University of Chicago, Chicago, IL 60637 USA; 4 Department of Dermatology and Plastic Surgery, Faculty of Life Sciences, Kumamoto University, Honjo, Kumamoto Japan; 5 Committee on Cancer Biology, University of Chicago, Chicago, IL 60637 USA; 6 Committee on Immunology, University of Chicago, Chicago, IL 60637 USA

**Keywords:** Recombinant protein therapy, Tissue engineering

## Abstract

Non-healing wounds have a negative impact on quality of life and account for many cases of amputation and even early death among patients. Diabetic patients are the predominate population affected by these non-healing wounds. Despite the significant clinical demand, treatment with biologics has not broadly impacted clinical care. Interleukin-4 (IL-4) is a potent modulator of the immune system, capable of skewing macrophages towards a pro-regeneration phenotype (M2) and promoting angiogenesis, but can be toxic after frequent administration and is limited by its short half-life and low bioavailability. Here, we demonstrate the design and characterization of an engineered recombinant interleukin-4 construct. We utilize this collagen-binding, serum albumin-fused IL-4 variant (CBD-SA-IL-4) delivered in a hyaluronic acid (HA)-based gel for localized application of IL-4 to dermal wounds in a type 2 diabetic mouse model known for poor healing as proof-of-concept for improved tissue repair. Our studies indicate that CBD-SA-IL-4 is retained within the wound and can modulate the wound microenvironment through induction of M2 macrophages and angiogenesis. CBD-SA-IL-4 treatment significantly accelerated wound healing compared to native IL-4 and HA vehicle treatment without inducing systemic side effects. This CBD-SA-IL-4 construct can address the underlying immune dysfunction present in the non-healing wound, leading to more effective tissue healing in the clinic.

## Introduction

The immune system’s involvement in many disease pathologies has become increasingly more relevant, and regeneration and wound repair are no exception^1–5^.

Healthy wound healing is a complex and well-orchestrated process that depends on the initiation and cessation of multiple signals as the wound environment moves through each phase of healing^6–9^. The first phase includes initial coagulation to stop bleeding followed by inflammation which serves to clear invading bacteria via neutrophil infiltration and clearing subsequent apoptotic debris through macrophage-mediated phagocytosis^10,11^. After the wound bed is clear of debris, the wound can progress to the proliferative and remodeling phases, in which blood vessel formation and extracellular matrix (ECM) deposition occur^12^. Many components of a non-healing wound are highly dysfunctional and stray from this path in many ways. The altered immune profile within non-healing wounds, when compared to that of healing wounds, is one example of this dysfunction^13–15^. The diabetic disease state is well-known for inducing non-healing wounds and altered immune profiles have been implicated in the etiologies of these non-healing wounds. For example, non-healing diabetic wounds exhibit both decreased and delayed neutrophil and macrophage recruitment, creating a non-productive inflammatory environment where the inflammatory signals in the wound bed are insufficient to serve out their function and ultimately halt healing and create a chronic inflammatory environment^16^. One mechanism of immune dysfunction is the dysregulation of soluble cytokine signals present within the milieu when compared to healthy, healing wounds^16^. These differences present the opportunity to treat non-healing wounds through immunotherapy, specifically therapeutically administered immune cytokines to modulate the immune microenvironment within the dysfunctional diabetic wound and improve therapeutic outcomes through restoration of the healthy healing environment. Through this work we engineered a cytokine construct that modulates the microenvironment of non-healing diabetic wounds. This strategy could be utilized with a variety of payloads in several diseases associated with dysfunctional dermal immunological profiles.

Within the context of non-healing wounds, growth factor therapy have been studied as a promising strategy, but showed limited clinical utility due to safety concerns and suboptimal efficacy^6,17^. For example, recombinant human VEGF-A was in Phase II clinical trials, but improvement of diabetic foot ulcer healing was marginal after topical application^18,19^. Recombinant PDGF-BB showed promise in efficacy but concerns regarding risk of cancer associated with its use have been raised^20,21^. Engineering strategies have been used to target these growth factors to the extracellular matrix (ECM), to minimize adverse effects when used to treat diabetic wounds^22,23^. In this fashion, but with an immune cytokine rather than a growth factor, we designed an IL-4 variant targeted to ECM in the non-healing wound microenvironment. One overarching concern that is applicable to protein-based therapies is degradation by proteases in the wound environment, a reason for the limited efficacy of growth-factor based therapies that could impact cytokine-based therapies as well.

Interleukin-4 (IL-4) is a potent cytokine that has many functions and has garnered interest as a cytokine that can facilitate regeneration of many tissue types^24–26^. Interleukin-4 signals through the phosphorylation of STAT6, leading to downstream changes in target cell phenotype. One example of this is the polarization of macrophages away from the pro-inflammatory associated phenotype (M1) and towards a phenotype that promotes tissue healing (M2)^27,28^. This shift in macrophage phenotype is crucial for a wound to move from the inflammatory phase of wound healing onward to the ECM deposition and regeneration phase^29^. Notably, in the diabetic wound, the ratio between M1 and M2 macrophages is skewed towards M1 compared to healthy, healing skin^30^. Though macrophage activation states are considered more of a spectrum than a clear-cut line^31,32^, this general categorization strategy is suitable within this context. This M1/M2 imbalance has been recapitulated in a murine type 2 diabetic model (*db/db)*, as db/db mice demonstrate reduced expression of M2-relate genes such as Ym1 and Arg-1 and elevated expression of M1 related genes such as iNOS^30^. These activation states carry over into human biology. In samples of non-healing wounds from type 2 diabetic patients, macrophages present in the wound resemble the classically activated M1 phenotype rather than pro-regenerative M2 macrophages^33–36^. This M1/M2 macrophage imbalance is implicated in other instances of chronic inflammation and inflammatory disorders such as pressure ulcers, arterial insufficiency ulcers, and venous leg ulcers^37–39^. M2 macrophages have been of primary interest with regards to non-healing wounds as they are critical to wound repair as they are thought to potentiate wound repair through their secretion of anti-inflammatory cytokines, facilitation of blood vessel formation, and deposition of ECM^40–42^. This imbalance serves as an interesting target to promote wound healing by driving the phenotypic shift towards M2 macrophages in the non-healing wound microenvironment using IL-4. This strategy has been used in other models of chronic inflammation, such as multiple sclerosis, and showed a therapeutic effect in these models, but to our knowledge has not been used for the treatment of non-healing wounds^43^. Still, current difficulties for unmodified cytokines as therapeutics are primarily related to lack of efficacy due to their short-lived bioavailability due to quick clearance from the bloodstream and their off-target effects, as many cytokine effects are highly dependent on what other signals are present^44^. These features have limited the clinical translation of cytokines as therapeutics^45^. A cytokine engineering strategy that can leverage spatiotemporal control of immune cytokine signaling to enhance desirable effects would be significant and beneficial for the utilization of cytokines in the therapeutic context.

To address these concerns, we hypothesized that exposed collagen within the wound bed would make a suitable target for cytokine binding after local administration. Collagen is highly abundant in the dermal tissue as a primary component of the ECM and is highly exposed in a wound, making it a practical target^46,47^. As a collagen-binding domain (CBD), we fused the A3 domain of von Willebrand factor, which binds both collagen I and collagen III^48,49^. Further, serum albumin (SA), a well characterized and abundant protein in the body, was added to increase the molecular weight of the final construct to improve retention within the hydrogel composition. Here, we show that IL-4 fused to both SA and the A3 domain, creating a CBD-SA-IL-4 recombinant protein, exhibits the desired polarization effects on macrophages as well as favorable spatiotemporal control when topically administered to cutaneous wounds in a hyaluronic acid (HA)-based hydrogel. This work thus demonstrates the benefit of engineered cytokine constructs, in particular IL-4, for inducing skin regeneration, using a model of type 2 diabetic wound healing as a proof-of-concept.

## Results

### CBD-SA-IL-4 binds to collagen, induces M2 macrophages in vitro, and is retained within wounds in vivo

**Fig. 1:**
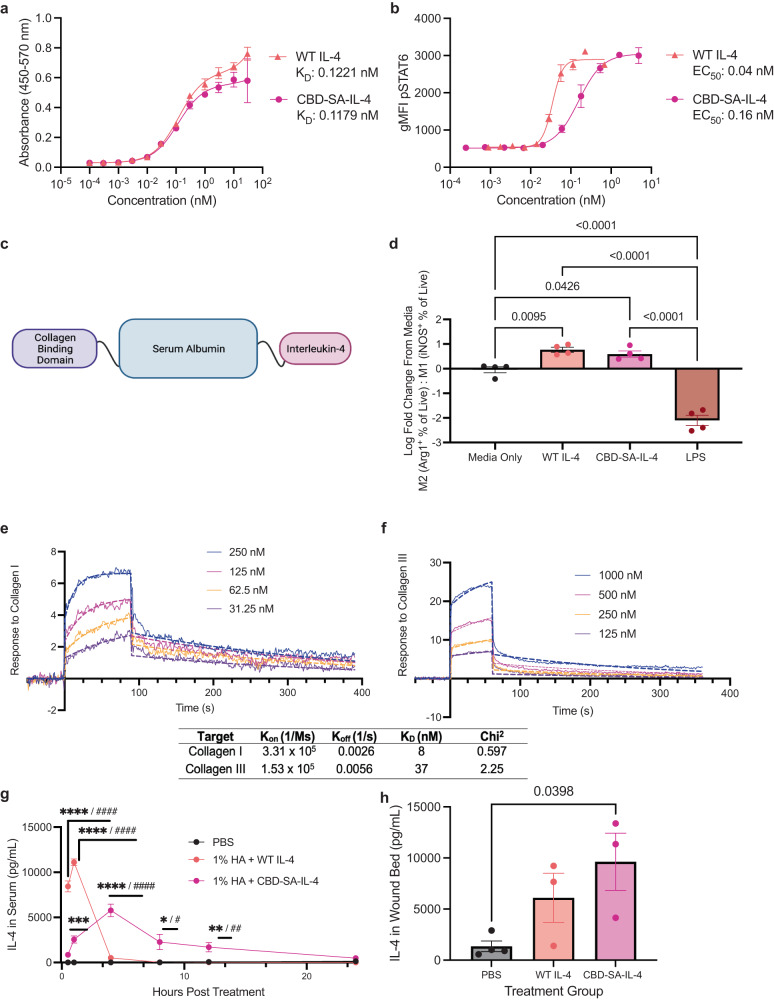
CBD-SA-IL-4 protein binds to collagen and is retained in the wound tissue when topically applied. **a** ELISA using immobilized IL-4Rα (*n* = 2; mean ± SEM). **b** Dose-dependent signaling through STAT6 in RAW264.7 cell line with both WT IL-4 and engineered construct CBD-SA-IL-4 (*n* = 2, mean ± SEM). EC_50_ values shown. **c** Schematic depicting composition of engineered IL-4 construct, flexible linkers are composed of (GGGS)_2_ sequence. **d** Polarization of RAW264.7 macrophage-like cell line towards M2 phenotype (Arg1^+^) when treated with WT IL-4 or CBD-SA-IL-4 (65 ng/mL IL-4 equivalent) or M1 (iNOS^+^) when treated with LPS (50 ng/mL) (*n* = 4; mean ± SEM). **e** Affinities (K_D_ values are shown) of CBD-SA-IL-4 against collagen I and (**f**) collagen III were measured by SPR. A SPR chip was functionalized with collagen I (**e**) or collagen III (**f**) and the engineered construct was flowed over the chip at indicated concentrations. Curves represent the specific responses in (RU) to each collagen observed. Experimental curves were fitted with Langmuir binding kinetics. **g** Release of CBD-SA-IL-4 or WT IL-4 (100 µg/ mL IL-4 equivalent) from 1% HA carrier after topical application in vivo, IL-4 concentration from serum timepoints quantified by IL-4 ELISA, using appropriate standard for each construct to account for binding differences (*n* = 3; mean ± SEM). **h** IL-4 content in the wound microenvironment quantified by IL-4 ELISA, using appropriate standard for each construct to account for binding differences 24 hr after treatment with IL-4 constructs containing HA gels in vivo (*n* = 3; mean ± SEM). Statistics: **d** Analyzed using ordinary one-way ANOVA and Tukey correction for multiple comparisons. **g** Analyzed using ordinary one-way ANOVA with Tukey corrected multiple comparison against all groups for each time point (* against PBS, # against IL-4 group). **h** Analyzed using ordinary one-way ANOVA with Tukey corrected multiple comparison against all groups. *p < 0.05, **p < 0.01, ***p < 0.001.

We produced and purified a CBD-SA-IL-4 recombinant fusion protein in mammalian cells and characterized it in vitro. Both WT IL-4 and the CBD-SA-IL-4 fusion protein bind the IL-4Rα subunit with equal affinity (Fig. 1a). Like its wild type (WT) counterpart, CBD-SA-IL-4 induced phosphorylation of STAT6 in a dose-dependent manner, but with a resulting EC_50_ 40-fold less than WT IL-4 (Fig. 1b). Based upon these results, the difference in signaling capacity is likely due to steric effects of the large serum albumin fusion reducing binding of the IL-4/IL-4Rα complex to the common gamma chain subunit of the receptor. Because IL-4 is known to induce an M2 phenotype in macrophages^50–52^, we cultured RAW264.7 macrophage-like cells with CBD-SA-IL-4 and its wild type counterpart and found that CBD-SA-IL-4 and WT IL-4 similarly polarized macrophages toward the M2 phenotype, as demonstrated by an increase in Arg1^+^ cells. As a control for the M1 phenotype, we included lipopolysaccharide (LPS) in the experimental panel, which induced the upregulation of iNOS (Fig. 1d). CBD-SA-IL-4 protein also demonstrated a high affinity for both collagen I and III, as quantified as the equilibrium dissociation constant determined via surface plasmon resonance (SPR), showing a K_D_ = 8 nM for collagen I and a K_D_ = 37 nM for collagen III (Fig. 1e, f). The high binding affinity is on a similar order to that observed in our previous work with CBD fused to other cytokines^49^.

We then incorporated CBD-SA-IL-4 protein into a simple hydrogel that can be applied topically to the wound. HA is a well-studied, clinically used and biologically active polymer that has been shown to regulate tissue repair and is also considered safe; thus, we selected it to complement the effects of the CBD-SA-IL-4^53–56^. Previously, HA-based dressings have been used as carriers for cellular therapies such as autologous fibroblasts^57^. We selected a 1% (w/v) composition of HA as our carrier based upon its desirable viscoelasticity that allowed easy topical application of the treatment without running out of the wound. To understand the release of CBD-SA-IL-4 from the 1% HA gel in vivo, we wounded C57BL/6 J mice and applied a 1% (w/v) HA gel containing either WT IL-4 or CBD-SA-IL-4. We collected blood at 0.5, 1, 4, 8, 12, and 24 h to look for systemic IL-4, and we homogenized the wounds at 24 h to look for IL-4 remaining in the treatment site. We demonstrated that significantly less CBD-SA-IL-4 entered the systemic circulation when compared to the WT IL-4 (Fig. 1g), as quantified by an IL-4 ELISA of the serum. Additionally, after 24 h there was more IL-4 present in the wounds treated with CBD-SA-IL-4 when compared to the WT IL-4 (Fig. 1h). These data show that the engineered CBD-SA-IL-4 construct displays slower systemic release kinetics compared to WT IL-4. The data also demonstrate that more CBD-SA-IL-4 is retained in the wound microenvironment as compared to WT IL-4, creating a localized effect while minimizing systemic exposure.

### CBD-SA-IL-4 therapy accelerates healing in type 2 diabetic non-healing wound model

**Fig. 2:**
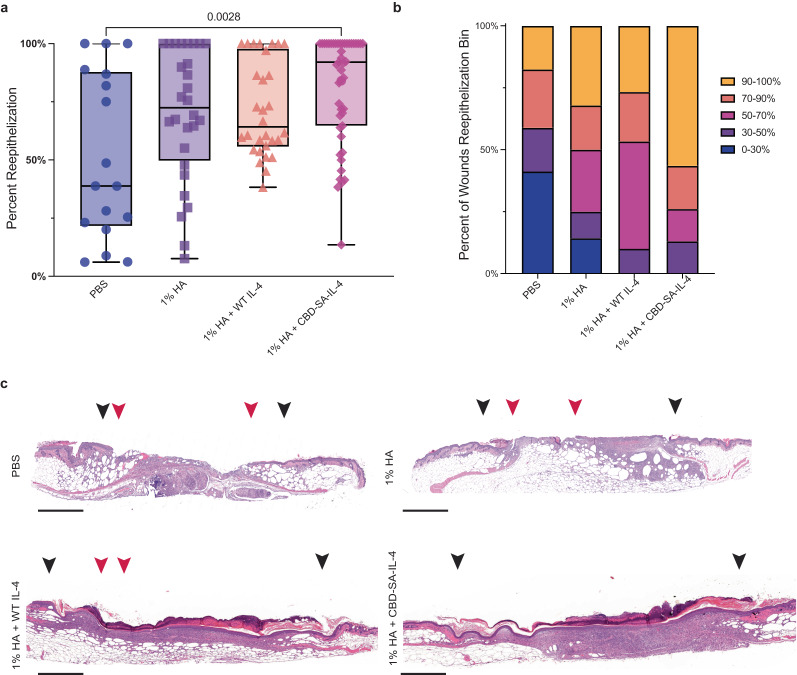
Topically applied 1% HA + CBD-SA-IL-4 enhances skin wound healing. Full-thickness back-skin wounds in 8-10-week-old C57BLKS/J-m/Lepr db (db/db) male mice treated with CBD-SA-IL-4 (100 µg/ mL) or WT IL-4 (100 µg/ mL). Four treatment groups were tested: PBS, 1% HA, 1% HA + WT IL-4, and 1% HA + CBD-SA-IL-4. After 11 days, **a**, **b** extent of reepithelization was evaluated by histology (PBS: *n* = 17, mean = 52.37% ± SEM); (1% HA *n* = 28, mean = 69.90 ± SEM); (1% HA + WT IL-4 *n* = 30, mean = 71.87 ± SEM); (1% HA + CBD-SA-IL-4 *n* = 47, mean = 81.29 ± SEM). **b** The wounds were binned based on percent closure after 11 days of healing (i.e. what percent of wounds were 90%–100% re-epithelized). **c** Representative wound histology (hematoxylin and eosin staining) at day 11 (scale bar 800 μm). Black arrowheads indicate margin of wound and red arrowheads indicted the tips of epithelium tongue. Statistics: **a** Analyzed using nonparametric, Kruskal–Wallis test with Dunn’s multiple comparison of mean rank of each group against the 1% HA + CBD-SA-IL-4 treated group. *p < 0.05, **p < 0.01, ***p < 0.001. Pooled from 3 experimental replicates. The non-parametric test was used due to the non-normality of the data set as confirmed by an Anderson–Darling test.

We tested a topically-delivered CBD-SA-IL-4 carried in a 1% (w/v) HA gel in PBS applied four days after surgically inflicted wounds in the type-2 diabetic db/db mouse, which is a well-established and clinically relevant model of impaired wound healing^22,58^. To more closely approximate human healing, we applied a silicone splint around each wound to prevent closure by skin contraction, the primary form of wound closure in mice but not humans. We compared four treatment groups: PBS, 1% HA, 1% HA + WT IL-4, and 1% HA + CBD-SA-IL-4. The PBS group represented an untreated wound, while the 1% HA group served as a carrier-only control, though due to its use in clinic already, we expected that it might help promote some healing due to the retention of moisture within the wound environment. We also expected wounds treated with 1% HA + WT IL-4 to show improved healing, but due to its quick clearance and leakage into the periphery likely would be less clinically relevant than our localized engineered therapy. Wounds were excised and analyzed via H&E staining 11 days after wounding, thus 7 days after treatment. As a result, 1% HA + CBD-SA-IL-4 treatment significantly promoted reepithelialization when compared to the PBS-treated wounds (Fig. 2a–c). Strikingly, 56% of wounds treated with 1% HA + CBD-SA-IL-4 showed >90% reepithelialization. In contrast, only 18%, 32%, and 27% of PBS, 1% HA, and 1% HA + WT IL-4 treated wounds had reepithelialization above 90%, respectively (Fig. 2b). 1% HA or 1% HA + WT IL-4 did not enhance reepithelialization compared to PBS treatment. To further assess the degree of wound closure, granulation tissue area of these wounds was quantified. These results demonstrate increased granulation tissue formation in both IL-4 treated groups (Supplementary Fig. 1a). Additionally, the reepithelialization seen in the H&E images was recapitulated with cytokeratin 16 IHC analysis, further confirming the extent of epithelialization (Supplementary Fig. 1b, c). Furthermore, collagen deposition and vasculature were confirmed in the 1% HA + CBD-SA-IL-4 treatment group via Masson trichrome staining and CD31 IHC, respectively (Supplementary Fig. 2). Additionally, CBD-SA-IL-4 did not improve reepithelization of wounds in healthy skin (C57BL/6) (Supplementary Fig. 3). This further supports that CBD-SA-IL-4 rebalances the composition of soluble signals and cellular populations present in specifically the diabetic wound environment. These data indicate that the topical treatment of 1% HA + CBD-SA-IL-4 improves wound closure when compared to the non-treated control, demonstrating that by modulating the signals present in the immune microenvironment of the wound, the dysregulation caused by the diabetic disease state can be modulated and therapeutic outcomes can be improved.

### CBD-SA-IL-4 induces macrophage recruitment to the wound microenvironment through soluble signals

**Fig. 3:**
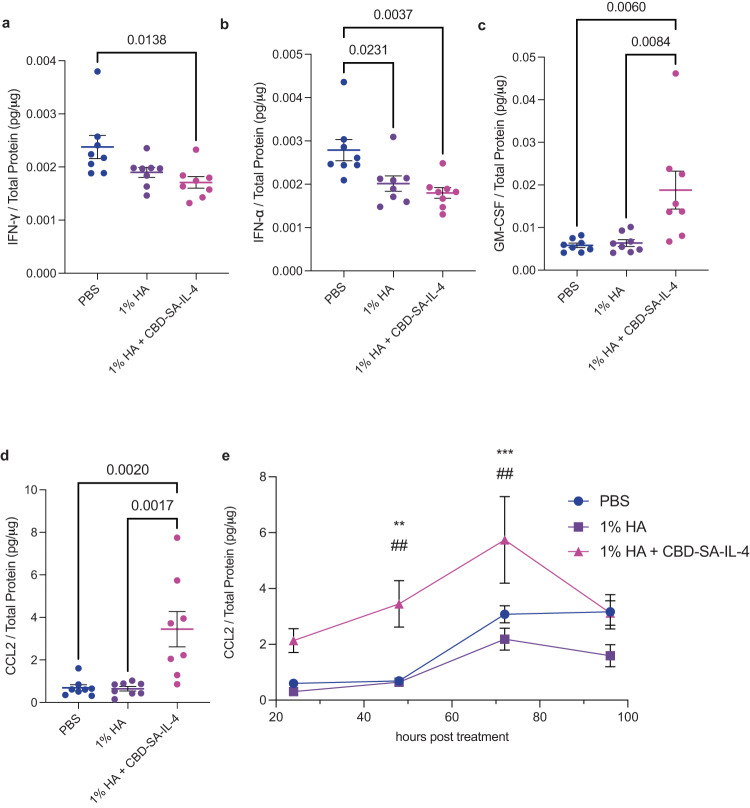
CBD-SA-IL-4 reduced inflammatory cytokines and induced regenerative cytokines/chemokines in the wounds. **a** IFN-α concentration in the wound 24 h post-treatment. **b** IFN-γ concertation in the wound 24 h post-treatment. **c** GM-CSF concentration in the wound 48 post-treatment. **d** CCL2 concentration in the wound 48 h post-treatment. **e** Kinetics of CCL2 concentration from 24, 48, 72 and 96 h post-treatment. Statistics: **a**–**d** Analyzed using ordinary one-way ANOVA and Tukey multiple comparison against all groups. **e** Analyzed using One-way ANOVA with Tukey corrected multiple comparison comparing treatment all treatment groups at each timepoint (* against 1% HA, # against PBS); *p < 0.05, **p < 0.01, ***p < 0.001 (all groups *n* = 8; mean ± SEM).

We next assessed the downstream mechanisms of action of CBD-SA-IL-4 on wound healing. We treated diabetic wounds following the same treatment timeline as above, excising wounds 24, 48, 72 and 96 h post-treatment. After 24 h, the wounds treated with either 1% HA and 1% HA + CBD-SA-IL-4 displayed significant decreases in both IFN-α and IFN-γ, indicating lower levels of inflammation within the wound microenvironment (Fig. 3a–b). Moreover, only wounds treated with CBD-SA-IL-4 showed significantly increased levels of granulocyte-macrophage colony stimulating factor (GM-CSF) when compared to both PBS-treated wounds and 1% HA-treated wounds (Fig. 3c). GM-CSF is a crucial regulator of granulocyte and macrophage lineage populations^59^ and promotes wounds healing^60^. Finally, CCL2, a chemoattractant for macrophages, demonstrated stark changes throughout all four timepoints, showing an initial increase at 24 and 48 h (Fig. 3d) post-treatment, peaking at 72 h, and finally returning to comparable levels to PBS-treated and 1% HA-treated wounds by 96 h post-treatment (Fig. 3e). This marked difference in CCL2 secretion follows the dynamic process we would expect with healthy healing, where macrophage recruitment occurs initially but quickly subsides^61^. These data show that 1% HA + CBD-SA-IL-4 induces signals to recruit macrophages to the wound.

### CBD-SA-IL-4 treatment increases M2 macrophages and pro-angiogenic cell populations in the wound

**Fig. 4:**
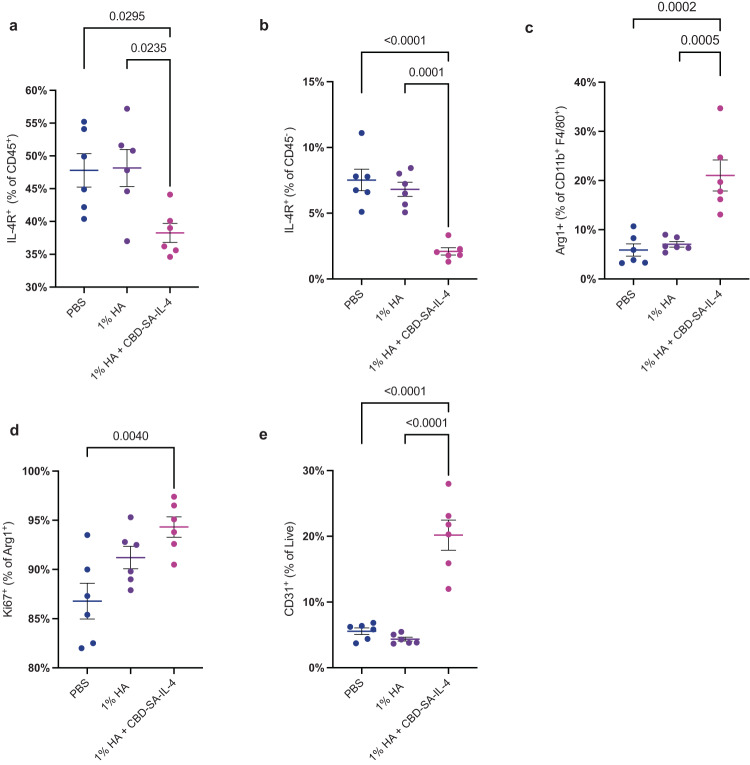
CBD-SA-IL-4 (100 µg/ mL) induces M2 macrophage proliferation and angiogenesis in the wounds. **a** IL-4Rα^+^ cells as a percent of CD45^+^ cells. **b** IL-4Rα^+^ cells as a percent of CD45− cells. **c** M2 macrophages (Arg1^+^) as a percentage of total macrophages (CD11b^+^ F4/80^+^). **d** Proliferative (Ki67^+^) M2 macrophages as a percentage of total M2 macrophages. **e** Angiogenic cells (CD31^+^) as a percentage of live cells. Statistics: **a**–**e** Ordinary one-way ANOVA with Tukey multiple comparison between all groups. *p < 0.05, **p < 0.01, ***p < 0.001 (all groups *n* = 6; mean ± SEM).

Because chemokine CCL2 expression was changed, we hypothesized that immune cell populations may have been altered by CBD-SA-IL-4 application. To understand how CBD-SA-IL-4 influences the cellular populations intra-wound, we treated diabetic wounds following the same treatment timeline as used previously, excising wounds 48 h post-treatment and digesting them to create single cell suspensions for flow cytometric analysis. Through this, we saw an increase in CD45^+^ infiltrating cells (Supplementary Fig. 4a). There was also a trend towards more macrophages (F480^+^ CD11b^+^) comprising the CD45^+^ infiltrates in the 1% HA + CBD-SA-IL-4 treated wounds when compared to PBS treated wounds (Supplementary Fig. 4b). Furthermore, there was a marked decrease in surface IL-4Rα expression on both CD45^+^ immune cells (Fig. 4a) and CD45^−^ non-immune cells (Fig. 4b) cell populations in the 1% HA + CBD-SA-IL-4 treated group as compared to the control groups, suggesting internalization of the receptor after IL-4 binding, which is a well-documented step of IL-4 signaling^62,63^. 1% HA + CBD-SA-IL-4 increased Arg1^+^ expression on macrophages, indicating their M2 phenotypic polarization, which was not seen in either the PBS− or 1% HA-treated groups (Fig. 4c). It is interesting to note that there was no difference in iNOS^+^ M1 macrophage populations when comparing all three groups. This finding could indicate the CBD-SA-IL-4 treatment is not polarizing the macrophages already present in the wound environment from M1 to M2 phenotype, but rather influencing the recruited macrophages and shifting their phenotype towards M2, however, the ratio between the M2 and M1 macrophages present was still favorably altered by the 1% HA + CBD-SA-IL-4 treatment (Supplementary Fig. 4c, d). Furthermore, the observed M2 macrophages showed increased Ki67^+^ expression, a marker of cellular proliferation, when treated with 1% HA + CBD-SA-IL-4 compared to PBS-treated wounds, indicating M2 macrophage proliferation in the wounds (Fig. 4d). Lastly, these M2 macrophages were associated with formation of new blood vasculature within the wound, as demonstrated by the increase in CD31^+^ cells in wounds treated with 1% HA + CBD-SA-IL-4 (Fig. 4e). These data show that 1% HA + CBD-SA-IL-4 modulates the cellular populations present within the wound environment and potentiates a more regenerative cellular phenotype.

### Single systemic administration of CBD-SA-IL-4 did not upregulate toxicity markers

**Fig. 5:**
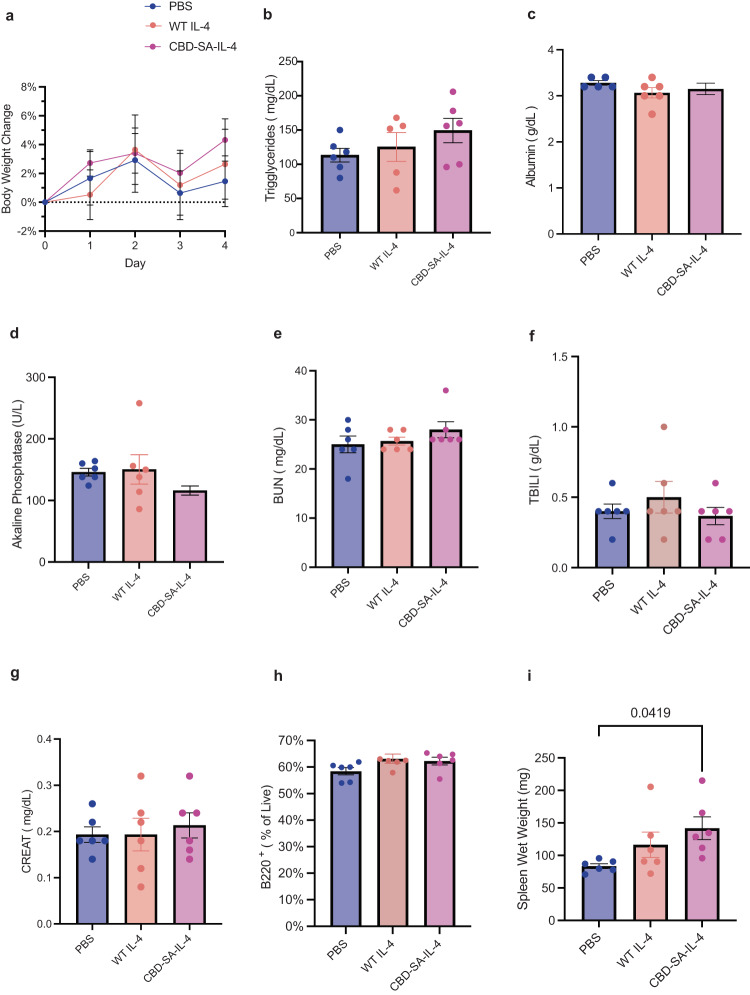
CBD-SA-IL-4 (100 µg/ mL IL-4 equivalent) does not show toxicity after a single subcutaneous administration. **a** Percent change in body weight over four-day time course. **b** Serum triglyceride levels two days post treatment. **c** Serum albumin levels two days post treatment. **d** Serum alkaline phosphatase level two days post treatment. **e** Serum blood urea nitrogen (BUN) levels two days post treatment. **f** serum total bilirubin (TBILI) levels two days post treatment. **g** Serum creatinine (CREAT) levels two days post treatment. **h** B Cells (B220^+^) in blood two days post treatment. **i** Spleen wet weight four days post-treatment. Statistics: Ordinary one-way ANOVA with Tukey multiple comparisons test was used. *p < 0.05, **p < 0.01, ***p < 0.001 (all groups *n* = 6; mean ± SEM).

Due to concerns of toxicity associated with cytokine therapy, we sought to explore toxicity of the locally administered CBD-SA-IL-4. While based on our measurements, we do not expect the entire dose to be systemically exposure after topical administration, to ensure a lack of toxicity in this worst case we administered a full dose subcutaneously. After subcutaneous administration of CBD-SA-IL-4 and WT IL-4 we monitored the body weight of healthy, wild-type mice for 4 days and demonstrated no changes in body weight (Fig. 5a). We analyzed organ damage markers in serum using a biochemistry analyzer to test whether CBD-SA-IL-4 induced any adverse effects in the blood two days post treatment. Treatment with CBD-SA-IL-4 did not change levels of organ-damage markers (Fig. 5b–g). Treatment with CBD-SA-IL-4 and WT IL-4 did increase spleen weight (Fig. 5h). Neither treatment induced a change in B cell counts in the blood, a documented phenomenon associated with prolonged IL-4 administration^64^. These data suggest that CBD-SA-IL-4 is well-tolerated after a single, systemic dose of 32 μg, reinforcing the safety of use at this concentration when applied topically to an open wound and systemic exposure is much less.

## Discussion

Current treatments for non-healing wounds primarily focus on management strategies and do not address the underlying dysfunctional and poorly regulated signaling milieu of the immune system^65,66^. Within the class of non-healing wounds, the immune system involvement and dysfunction had become better understood. The continued production of proinflammatory cytokines within the wound stagnates its repair and keeps the environment in a non-productive inflammatory stage, preventing progression to proliferation and remodeling^67,68^. In wound tissue in normal skin, there is an initial influx of immune cells, predominantly neutrophils and macrophages, and a subsequent resolution of these cells after debris has been cleared from the environment marked by a transition from M1 macrophages to M2 macrophages^69^. In the diabetic wound, the influx of these immune cells does not peak but rather plateaus, and inflammation remains insufficient to progress towards resolution^16,70,71^.

The soluble signals present within a wound microenvironment are crucial to the success of the healing process. Our engineered construct was able to modulate the immunologically active signals present in the wound environment toward a pro-regenerative profile^60,72,73^. Notably, the wounds treated with 1% HA + CBD-SA-IL-4 had significantly less IFN-α and IFN-γ when compared to the PBS treated wounds, demonstrating a suppression of general inflammation in the environment at 24-hr post treatment. Furthermore, within the context of cutaneous repair, CCL2 reportedly promotes healing in the diabetic wound through the restoration of the macrophage response. By pushing the wound environment out of the stagnant plateau of nonproductive inflammation it otherwise displays and by recruiting immune cells to the wound to clear debris and signal, the commencement of the subsequent phases^72^. The clear peak in CCL2 concentration within the wound microenvironment exhibited in the 1% HA + CBD-SA-IL-4 group indicates its ability to restore the influx of immune cells to achieve productive clearance of debris from the wound bed. Its subsequent decrease shows that this recruitment is resolved and does not continue, indicating the beginning of the resolution phase. Additionally, CCL2 has been shown to have angiogenic effects, and blocking CCL2 diminishes angiogenesis^74^, further supporting the benefits of wound repair of the induced CCL2 peak. Additionally, it has been demonstrated in diabetic patients that their non-healing wounds displayed significantly less GM-CSF when compared to healthy controls^60^. GM-CSF knockout mice demonstrate delayed wound healing, and it has been suggested that GM-CSF acts as keratinocyte proliferation inducer, beneficial for improving reepithelization^73,75^. The significant increase of GM-CSF displayed in the 1% HA + CBD-SA-IL-4 further indicates that our treatment is acting to restore the healthy healing environment through recruitment and biasing of the M2 macrophage population to the wound in a temporal manner. The signals present in the 1% HA + CBD-SA-IL-4 group thus demonstrate a molecular shift that could benefit wound healing when compared to both the PBS and 1% HA treated groups.

The differences in molecular signals described above influence the changes in cellular phenotype we see between treatment groups, as demonstrated through flow cytometry of the wound tissue early in the healing trajectory. It has been shown in the literature that cytokine receptors are internalized after binding of their ligand, and this is the case for IL-4Rα and IL-4^62,63^. With this considered, our data show that IL-4 treatment is active and is acting on both CD45^+^ and CD45^-^ cell populations in the wound as indicated by the decrease in IL-4Rα expression ultimately showing that, despite having reduced pSTAT6 activity in vitro, the functionality of the construct is retained. The wounds treated with 1% HA + CBD-SA-IL-4 also showed a striking increase in the frequency of M2 macrophages when compared to both the PBS-treated and 1% HA-treated groups. This strong increase in M2 macrophages indicates a shift caused by the 1% HA + CBD-SA-IL-4 towards a regenerative cellular phenotype. Additionally, 1% HA + CBD-SA-IL-4 treatment induces an increase in CD31^+^ cells within the wound, a population that has demonstrated the ability to induce blood vessel formation in disease models such as ischemic vascular disease^76^. This is especially pertinent in the healing of non-healing wounds where blood vessel formation is stunted and a hypoxic environment persists, which is thought to be an important contributor to dysregulated healing both diabetic and non-diabetic ulcers^77–79^. It has also been shown that IL-4Rα signaling in myeloid cells directly controls collagen fibril assembly in the skin^40^. Overall, our data indicate that IL-4 treatment induces cellular changes that strongly indicate a pro-regenerative environment through polarization of macrophages and formation of blood vasculature, which ultimately result in improved reepithelization.

It is interesting to consider the work done by Zhao and colleagues, where chronic expression of IL-4 was related to aberrant wound healing in non-diabetic mice^80^. The results seen in that study, which show increased CCL2 expression and more cellular infiltrates to the wound, fall in line with the findings we lay out here. However, there are two key considerations to contextualize that account for the improved healing we see in our work and the poor wound healing seen in this study. The first aspect to consider is the animal model used by Zhao is that of a wild type mouse, and therefore has a differing baseline milieu in the wound from the work we depict here. Secondly, the IL-4 signal we provide to the wound is not continuous, as it is in the work of Zhao. As discussed, the wound healing process is very tightly regulated in terms of temporal control, such that chronic IL-4 signaling may not provide the necessary cessation of signaling that allows one phase of healing to transition to the next. This is contrasted to our work, where the singular application of our IL-4 construct provides the required perturbation to the wound bed milieu to restore cellular infiltration and subsequent polarization but does not prolong this recruitment to detriment. Furthermore, it is important to consider the non-healing wound model used here and its limitations. The db/db mouse model, while commonly used, does not fully recapitulate human cutaneous healing and is highly variable^81^. Our work depicts a faster rate of healing in the control groups when compared to standard rates seen in mouse models of wound healing. There are many confounding factors that could alter the baseline rate of healing such as splinting methodologies, dressings and coverings or lack thereof, and microbiota constituencies^82–87^, which may account for variation in untreated wound healing between published reports. With this point considered, our design of CBD-SA-IL-4 is a exciting approach for the topical treatment of non-healing wounds, utilizing the exposed collagen within the dermal wound as an anchor to potentiate a localized therapeutic effect. Our engineering strategy could be applied to other immunologically relevant payloads to further manipulate the wound microenvironment to promote tissue repair.

This therapy differs from other strategies previously used in the field by targeting immune cells directly, with the intention of modulating the dysregulated immune profile present in non-healing wounds. Though it is reasonable to believe non-immune cells expressing the IL-4R, such as fibroblasts, also could be impacted by this treatment, our focus was centered on the myeloid population, specifically macrophages and their phenotype (either M1 or M2)^88–90^. While our data indicate increased M2 macrophages as being beneficial to healing of a diabetic wound, it is important to note that we did not observe a significant change in the M1 macrophage population. In a recent study conducted by Theocharidis and colleagues, it was found that ‘healing’ diabetic foot ulcers consisted of more M1 macrophages and fewer M2 macrophages than ‘non-healing’ diabetic foot ulcers^91^. Our findings, taken together with the findings of this work, could further support that not one cell type is beneficial, but rather the balance between cell types is more critical. Additionally, this study appears to depict less cellular infiltration into the ‘non-healing’ diabetic foot ulcer when compared to the ‘healing’ diabetic foot ulcer, a commonality between our findings and previous literature^91^.

Previous attempts in development of a topical therapy have focused on growth factor-based treatment, which are a downstream result of earlier signals present at large within the diabetic wound microenvironment^92^. Additionally, growth factor therapies have historically been associated with higher risk of cancer induction and have seen limited clinical success due to such safety and toxicity concerns, as well as susceptible to proteolytic degradation^20,21,93^. Through the protein engineering strategies previously discussed, CBD-SA-IL-4 allows for a localized immunotherapy that minimizes off-target, systemic effects. Our toxicity analyses showed that treatment with CBD-SA-IL-4 is well-tolerated. Although we observed modest splenomegaly, this is typically transient and not considered as a critical toxicity; moreover, this was observed with subcutaneous administration as a surrogate for the worst-case of topical administration. Our work here serves as a proof-of-concept for topical IL-4 based therapy, which can potentially address the highly unmet clinical need of non-healing wounds, most commonly diabetic foot ulcers, where currently 30% of diabetic patients undergo amputation due to these non-healing wounds and, the 5-year overall survival rate following an amputation in diabetic patients is only 43%^94–96^ In conclusion, here we show that our engineered IL-4 construct, CBD-SA-IL-4, can bind collagen in a non-healing wound microenvironment, and through the polarization of macrophages towards a pro-regenerative M2 phenotype, can facilitate reepithelization of a skin wound in the db/db mouse model of Type 2 diabetes.

## Methods

### Cytokine production and purification

The sequence encoding for CBD-SA-IL-4 was synthesized and subcloned into the mammalian expression vector pcDNA3.1(+) by Genscript, the amino acid sequence is listed in the supplemental materials (Supplementary Table 1). A sequence encoding for 6 His was added at the N-terminus for affinity purification of the recombinant protein. Suspension-adapted HEK-293F (Gibco) cells were routinely maintained in serum-free FreeStyle 293 Medium (Gibco). On the day of the transfection, cells were inoculated into fresh media at a density of 1 × 10^6^ cells/mL. A total of 1 µg/mL plasmid DNA, 2 µg/mL linear 25-kDa polyethylenimine (Polysciences), and OptiPRO SFM media (4% final concentration, ThermoFischer) were sequentially added. The culture flask was agitated by orbital shaking at 135 rpm at 37 °C in the presence of 5% CO_2_. Six days after transfection, the cell culture medium was collected by centrifugation and filtered through a 0.22 µm filter. Culture media was loaded into a HisTrap HP 5-mL column (GE Healthcare), using and ÄKTA Pure 25 instrument (GE Healthcare) as done previously^48^. After binding of the protein with binding buffer (20 nM NaH_2_PO_4_, 0.5 M NaCl, pH 7.4) protein was eluted with stepwise increases of 500 mM imidazole (in 20 nM NaH_2_PO_4_, 0.5 M NaCl, pH 7.4) (Supplementary Fig. 5A). The elution solution was further purified with size-exclusion chromatography using a HiLoad Superdex 200PG column (GE Healthcare) (Supplementary Fig. 5B). All purifications steps were carried out at 4 °C. The expression of cytokines was determined by a Nanodrop 2000 spectrophotometer. The proteins were verified as >90% pure by SDS-PAGE and images acquired with ChemiDoc XRS+ system (Bio-Rad) (Supplementary Fig. 5C). Constructs were confirmed to be free of endotoxin via a TLR4-Blue reporter cell line (Invitrogen).

### Bioactivity

RAW264.7 macrophage cell line (ATCC) were plated in 96 well non-tissue treated U-bottom plates at a concentration of 2 × 10^6^ cell/mL and allowed to adhere overnight. Stimulating media containing cytokine the construct was added in a serial dilution to the plate and incubated at 37 °C for 15 min. Cells were dissociated from the plate using an ice-cold solution containing PBS, 1 mM EDTA, and 1 mM EGTA. Phosflow Lyse/Fix Buffer (BD Biosciences) was added to the plate and incubated at 37 °C for 10 min. The plate was centrifuged and washed with PBS before adding Perm III Buffer (BD Biosciences) and incubating on ice for 30 min. The plate was centrifuged and washed with a 2% FBS solution in PBS. To block non-selective binding, anti-CD16/32 antibody (BioLegend) was applied and incubated for 15 min at room temperature. In a solution of 2% FBS in PBS PE anti-pSTAT6 antibody (BD Biosciences, 612701, 1:200) was added and incubated for 1 h at room temperature. Cells were analyzed using a Fortessa (BD Biosciences) flow cytometer and FlowJo software (FlowJo, LLC). Gating strategies are shown in the supplemental materials (Supplementary Fig. 6).

### Affinity

High binding 96 well plates were coated overnight at 4 °C with 10 nM IL-4Rα-Fc (R&D Systems). Wells were incubated with a titrated concentration of IL-4 or CBD-SA-IL-4, which was detected by a biotinylated anti-IL-4 antibody (ThermoFisher). Streptavidin-HRP enabled colorimetric quantification of bound IL-4 with the addition of TMB substrate (Millipore). Reaction was stopped with 2% H_2_SO_4_/1% HCl and absorbance was read at 450 nm.

## SPR

SPR measurements were performed as previously described^49^ using a Biacore X100 SPR system (GE Healthcare). Collagen I or collagen III (EMD Millipore) was immobilized by amine coupling on a CM5 chip (GE Healthcare) for around 1000 resonance units according to the manufacturer’s instructions. CBD–SA-IL-4 was flowed for 90 s (for collagen I) and 30 s (for collagen III) at increasing concentrations in the running buffer at 30 μl min−1. The sensor chip was regenerated with 50 mM NaOH for every cycle. Specific binding of CBD–SA-IL-4 to collagen was calculated automatically using the response to a non-functionalized channel as a reference. The binding curves were fitted using BIAevaluation (GE Healthcare). The binding results were fitted with Langmuir binding kinetics (1:1 binding with drifting baseline Rmax local).

### Macrophage polarization

RAW 264.7 (ATCC) cells were plated at a concentration of 2 × 10^6^ cells/mL in a 96-well U-bottom plate and allowed to adhere overnight at 37 °C. Cytokine-containing media was applied to the cells at a concentration of 65 ng/mL relative to IL-4 content and incubated for 24 h at 37 °C. Cells were dissociated using 1 mM EDTA and 1 mM EGTA in PBS. Nonspecific binding was blocked using anti-CD16/32 antibody (BioLegend). Cells were stained intracellularly for iNOS (Invitrogen, 58-5920-80, 1:200) and Arg1 (Invitrogen, 25-3697-82, 1:200). The eBiosciene Foxp3/Transcription Factor Staining Buffer Set kit was used and followed according to manufacturer’s instructions (Invitrogen). Cells were analyzed using a Fortessa (BD Biosciences) flow cytometer and FlowJo software (FlowJo, LLC). Gating strategies are shown in the supplemental materials (Supplementary Fig. 7).

### Mouse skin type 2 diabetic wound-healing model

Male C57BLKS/J-m/Lepr db (db/db) 8–10 weeks upon start of experimentation (The Jackson Laboratory) were used. Weights were recorded pre-operatively and found to be in line with vendor documentation with diabetic disease progression (Supplementary Fig. 8). At this age, the vendor has shown that 6% of mice are diabetic (https://www.jax.org/jax-mice-and-services/solutions-by-therapeutic-area/metabolic-diseases/featured-mice-for-type-2-and-obesity/phenotype-information-for-000642). Mice were given 0.1 mg/kg subcutaneous buprenorphine preemptively with a second dose administered 12 h post-operatively if needed. Their backs were shaved and cleaned and four full-thickness punch-biopsy wounds (6 mm diameter) were created in each mouse. Each wound was splinted open using a silicone ring (inner diameter 8 mm, outer diameter 12 mm) to prevent contraction. Four days post-surgery, PBS, hydrogel only, or cytokine containing hydrogels (80 µL in total, 100 µg / mL IL-4 based concentration) were topically applied to the wounds randomly. The wounds were covered with Adaptic dressing and sealed with adhesive film. Mice were single caged after the wounding surgery. After 11 days, mice were euthanized via CO_2_ inhalation, and the skin wounds were carefully excised for blinded histological analysis. All experiments using mice received approval from the Institutional Animal Use Committee of the University of Chicago under ACUP 72450. The animals’ care was in accordance with institutional guidelines. This experiment was repeated a total of three times for a final sample size of PBS (*n* = 17), 1% HA (*n* = 28), 1% HA + WT IL-4 (*n* = 30), 1% HA + CBD-SA-IL-4 (*n* = 47).

### Histomorphometric analysis of wound tissue sections

Wounds were fixed overnight in 2% PFA and cut down the center into two and embedded into paraffin for histological analysis on 5-µm serial sections. The extent of reepithelization was measured blindly through encoded file names by histomorphometric analysis of tissue sections (H&E stain) using QuPath software^97^. For analysis of reepithelization, the distance that the epithelium had traveled across the wound was measured; the muscle edges of the panniculus carnosus were used as an indicator for the wound boundary; and reepithelization was calculated as the percentage of the distance between the edges of the panniculus carnosus muscle. Enlarged images from Fig. 2 in supplemental materials (Supplementary Fig. 9). Raw photographs from Day 11 endpoint are also in the supplemental materials (Supplementary Fig. 10).

### Pharmacokinetics

Nine C57BL/6 J male mice aged 9-10 weeks (The Jackson Laboratory) were wounded using a 6 mm diameter biopsy punch to make a full thickness wound as described above. Cytokine-containing hydrogels at IL-4 equivalent concentrations of 100 µg / mL were immediately applied, and the wound was covered as described above. Blood was collected at relevant time points. Upon euthanasia, the wound tissue was excised in uniform area, 6 mm, and homogenized using T-Per Buffer (ThermoFisher) and Matrix D lysing tubes (MP Biomedical). Plasma was harvested in Heparin coated tubes and used for detection with pre-coated interleukin-4 ELISA kits (Invitrogen). An additional standard using recombinant CBD-SA-IL-4 to measure CBD-SA-IL-4 in plasma to account for impacted antibody binding.

### Cytokine profile of wound tissue

Male C57BLKS/J-m/Lepr db (db/db) 8–10-week-old mice (The Jackson Laboratory) were used and the same procedure was used as described above. Skin wounds were treated with hydrogels as described above. After 24, 48, 72 and 96 h, the wounded skin was removed as described above and transferred to T-Per Solution (ThermoFisher) in Lysing Matrix D containing tubes (MP Biomedical) and homogenized (MP Biomedical). Then, the solution was centrifuged, and the supernatant was retained for analysis using LegendPlex Mouse Cytokine Release Syndrome Multiplex Kit (BioLegend) carried out according to manufacturer’s instruction.

### Flow cytometric analysis of the wounds

Male C57BLKS/J-m/Lepr db (db/db) 8–10-week-old mice (The Jackson Laboratory) were used and the wounding procedure was performed in the same way as described above. Skin wounds were treated with hydrogels as described above. 48 h post treatment application, wounded skin was removed as described above and cut into small pieces (<5 mm^2^) and transferred to 4 mL of an enzyme solution (Liberase TL (0.4 mg/mL) Roche, DNase I (13.7 pU/mL) MP Biomedicals), and incubated for 2 h at 37 °C. Then, the cells from the digested wounds were resuspended in 25 mL of media, passed through a 70 µm cell strainer, and centrifuged. The single cell suspension was counted, and 2 × 10^6^ cells/mL were plated and stained for 15 min in 50 µL live/dead fixable dye and anti CD16/32 (BioLegend). After one wash cells were stained for 30 min in 50 µL of 2% FBS in PBS-containing antibodies (Table 1). Intracellular staining was performed using the eBiosciene Foxp3/Transcription Factor Staining Buffer Set according to the manufacturer’s instructions (Invitrogen). Cells were analyzed using an Aurora (Cytek) spectral flow cytometer and FlowJo software (FlowJo, LLC). Gating strategies are shown in the supplemental materials (Supplementary Fig. 11).

**Table 1:** Flow Cytometric Panel Design for Single Cell Wound Suspension.

Marker	Fluorophore	Catalog Number	Supplier	Clone	Dilution
CD86	BUV 395	564199	BD Horizon	GL1	50
CD31	BUV 563	741251	BD OptiBuild	MEC13.3	100
CD124	BUV 661	741557	BD OptiBuild	mIL4R-M1	50
CD45	BUV 805	752415	BD OptiBuild	13/2.3	200
CD206	BV 421	141717	BioLegend	C068C2	200
CD11b	BV 650	563402	BD Horizon	M1/70	400
CD11c	BV 711	117349	BioLegend	N418	100
F4/80	FITC	123108	BioLegend	BM8	400
Ly-6G	PerCP-Cy5.5	560602	BD Pharmagien	IA8	200
IA/IE	APC	107614	BioLegend	M51114.15.2	800
Ly-6C	APC-Fire 750	128045	BioLegend	HK1.4	400
SMA	Alexa Fluor 700	MBP2-34522AF700	Novus Bioscience	1A4/asm-1	100
Ki67	BV 605	652413	BioLegend	16A8	200
iNOS	Alexa Fluor 532	58-5920-80	Invitrogen	CXNFT	200
Arg1	PE-Cy7	25-3697-82	Invitrogen	AlexF5	200
Live Dead	Zombie NIR	423105	BioLegend	N/A	800

### Toxicological studies

Female C57BL/6 J 8-week-old mice (The Jackson Laboratory) were used. A single dose of either PBS, WT IL-4, or CBD-SA-IL-4 was administered subcutaneously at an equivalent concentration to that applied topically in previous wounding studies on all four wounds (32 μg). Body weight was monitored daily, and two days post dose administration blood was collected for analysis using a blood chemistry analyzer (Alfa Wassermann VetAxcel) and flow cytometric readouts. On day 4 post treatment, upon euthanasia, the spleen was harvested and weighed.

### Statistical analysis

Statistical methods were not used to predetermine the necessary sample size, rather sample sizes were chosen based on estimates from pilot experiments and previously published results such that appropriate statistical tests could yield significant results. Due to the high number of data points at the top of the dynamic range for the model (i.e. 100% healing) the dataset was considered non-normal. In order to confirm normality, an Anderson-Darling test was run. If the dataset was found to be non-normal, a nonparametric test would be run. *p < 0.05, **p < 0.01, ***p < 0.001.

## Material availability

Biological materials used are available upon reasonable request to the corresponding author.

### Reporting summary

Further information on research design is available in the Nature Research Reporting Summary linked to this article.

### Supplementary information

Supplemental Material

Reporting Summary

## Data Availability

The authors declare that all data supporting the results in this study are available within the paper and its Supplementary Information. The datasets generated and analysed during the study are available from the corresponding author upon reasonable request.

## Code availability

The QuPath software used for bioimage analysis is available at https://qupath.github.io/^97^.
