# Author Correction: Topically-applied collagen-binding serum albumin-fused interleukin-4 modulates wound microenvironment in non-healing wounds

**DOI:** 10.1038/s41536-023-00338-8

**Published:** 2023-11-02

**Authors:** Abigail L. Lauterbach, Rachel P. Wallace, Aaron T. Alpar, Kirsten C. Refvik, Joseph W. Reda, Ako Ishihara, Taryn N. Beckman, Anna J. Slezak, Yukari Mizukami, Aslan Mansurov, Suzana Gomes, Jun Ishihara, Jeffrey A. Hubbell

**Affiliations:** 1 Pritzker School of Molecular Engineering, University of Chicago, Chicago, IL 60637 USA; 2 Department of Bioengineering, Imperial College London, London, W12 0BZ UK; 3 Committee on Molecular Metabolism and Nutrition, University of Chicago, Chicago, IL 60637 USA; 4 Department of Dermatology and Plastic Surgery, Faculty of Life Sciences, Kumamoto University, Honjo, Kumamoto Japan; 5 Committee on Cancer Biology, University of Chicago, Chicago, IL 60637 USA; 6 Committee on Immunology, University of Chicago, Chicago, IL 60637 USA

**Keywords:** Recombinant protein therapy, Tissue engineering

Correction to: *npj Regenerative Medicine* 10.1038/s41536-023-00326-y, published online 11 September 2023

In this article, the QuPath software was not properly cited and should have included the following citation: Bankhead, P. et al. QuPath: open source software for digital pathology image analysis. *Sci. Rep*. **7**, 16878 (2017). 10.1038/s41598-017-17204-5.

The Code Availability sentence should have read, 'The QuPath software used for bioimage analysis is available at https://qupath.github.io/^97^.

The original article has been corrected.

