# Supplementary material for: Topically-applied collagen-binding serum albumin-fused interleukin-4 modulates wound microenvironment in non-healing wounds
Source: NPJ Regen Med. 2023 Sep 11;8:49. doi: 10.1038/s41536-023-00326-y (PMC10495343; doi:10.1038/s41536-023-00326-y)
Supplement: Supplementary file 1 — Supplemental Material [file 41536_2023_326_MOESM1_ESM.pdf]

Topically-Applied Collagen-Binding Serum Albumin-Fused Interleukin-4 Modulates Wound Microenvironment in Non-Healing Wounds

Abigail L. Lauterbach[1], Rachel P. Wallace[1], Aaron T. Alpar[1], Kirsten C. Refvik[1], Joseph W. Reda[1], Ako Ishihara[1,2], Taryn N. Beckman[3], Anna J. Slezak[1], Yukari Mizukami[4], Aslan Mansurov[1], Suzana Gomes[1], Jun Ishihara[1,2*], & Jeffrey A. Hubbell[1,5,6*]

1. Pritzker School of Molecular Engineering, University of Chicago, Chicago, Illinois 60637, United States
2. Department of Bioengineering, Imperial College London, London, United Kingdom, W12 0BZ
3. Committee on Molecular Metabolism and Nutrition, University of Chicago, Chicago, Illinois 60637, United States
4. Department of Dermatology and Plastic Surgery, Faculty of Life Sciences, Kumamoto University, Honjo, Kumamoto, Japan
5. Committee on Cancer Biology, University of Chicago, Chicago, Illinois 60637. United States
6. Committee on Immunology, University of Chicago, Chicago, Illinois 60637, United States

*Corresponding authors: j.ishihara@imperial.ac.uk, jhubbell@uchicago.edu

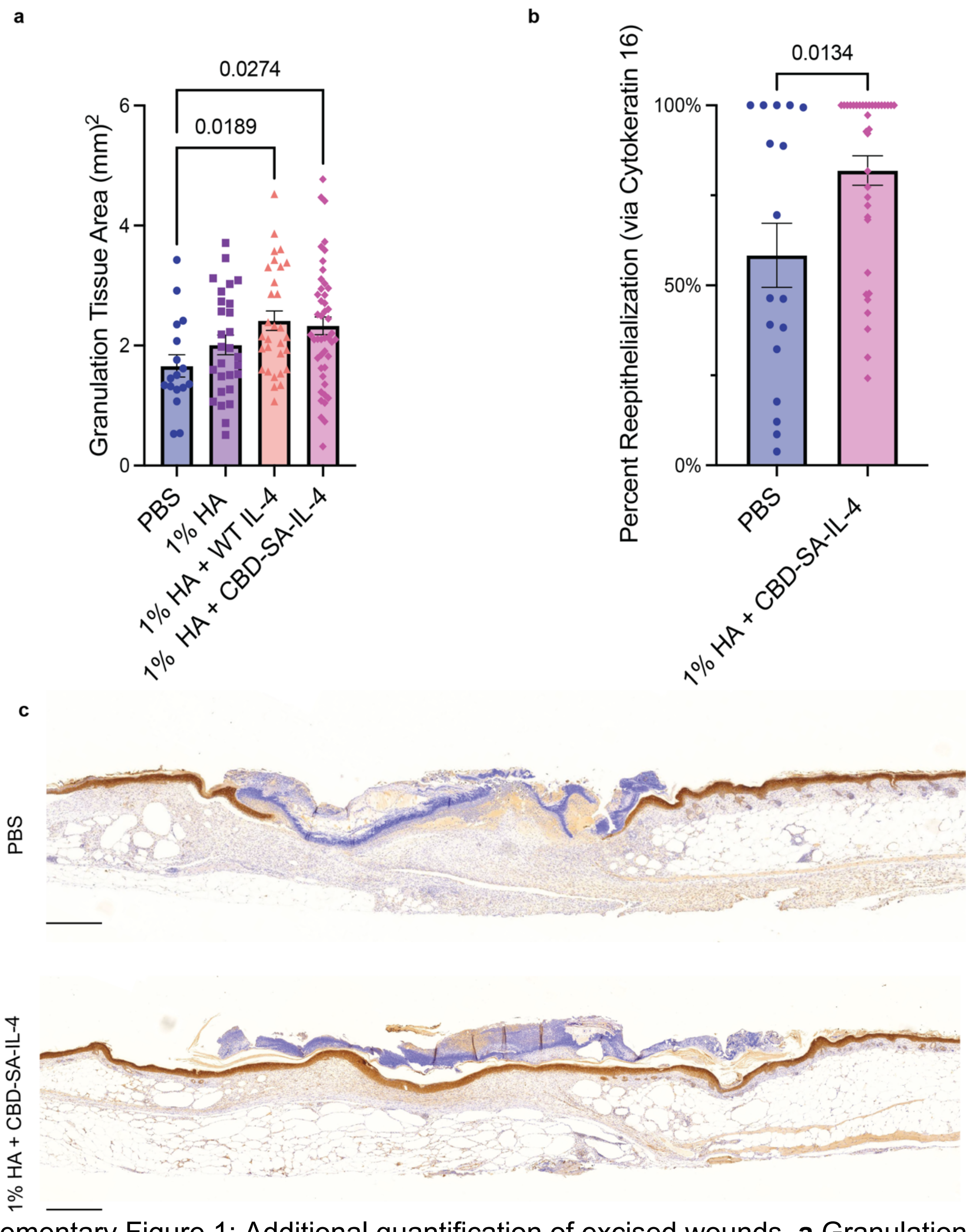

Supplementary Figure 1: Additional quantification of excised wounds. **a** Granulation tissue area quantification. **b** Percent reepithelization quantified through cytokeratin 16 IHC immunostaining. **c** Representative cytokeratin 16 IHC immunostaining images for PBS and 1% HA + CBD-SA-IL-4. Scale bar 500 µm. Statistics: **a** Analyzed using ordinary one-way ANOVA and Dunnett multiple comparison against PBS **b** unpaired t-test Mann-Whitney test; * p<0.05, **p<0.01, *** p<0.001. Pooled from 3 experimental replicates.

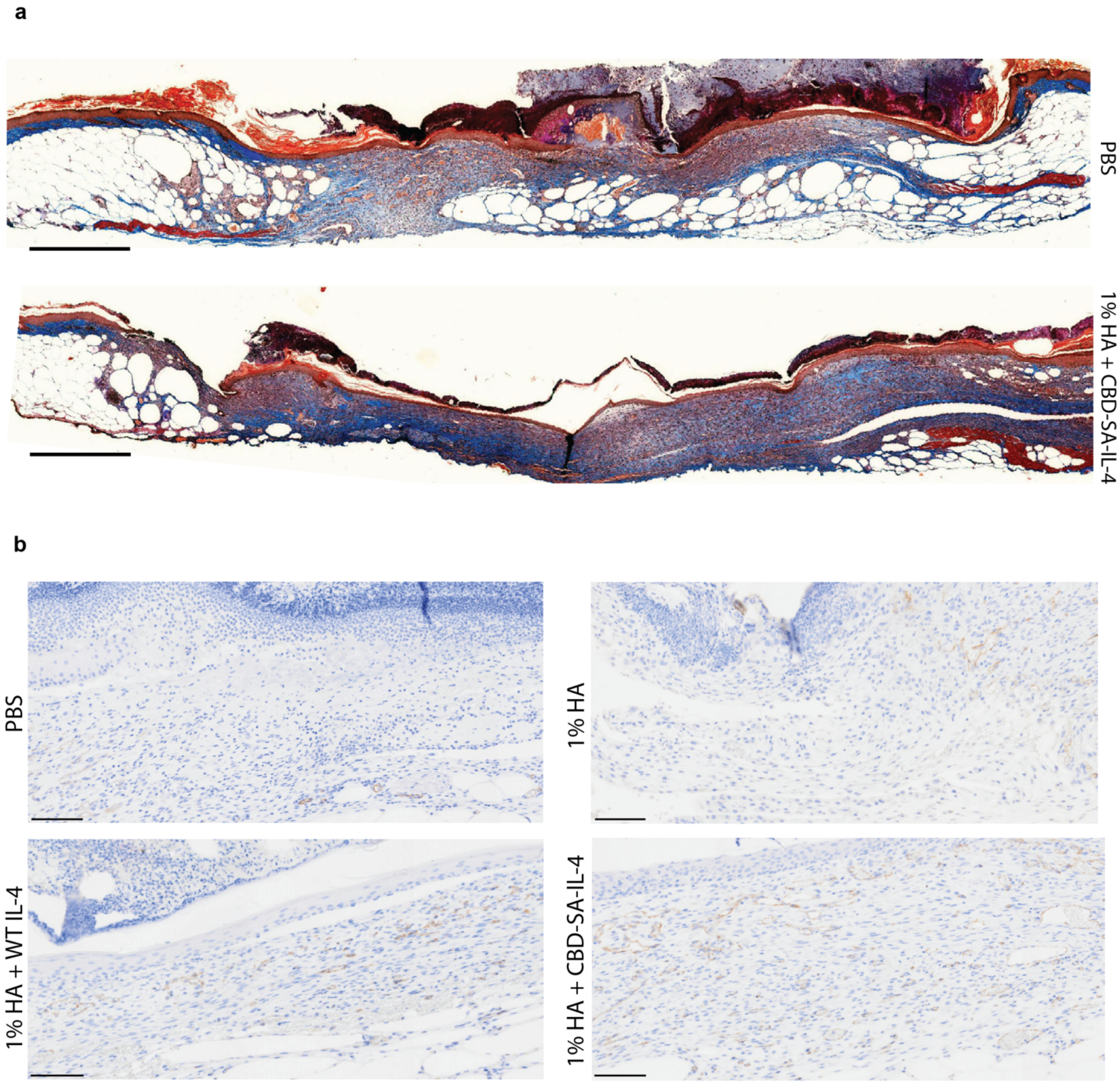

Supplementary Figure 2: Qualitative histology visualizing collagen deposition and vasculature. **a** Masson trichrome staining of PBS and 1% HA + CBD-SA-IL- treated wounds, blue coloring represents collagen deposition. Scale bar 600 µm **b** Representative CD31 IHC stained wound sections. Scale bar 100 µm.

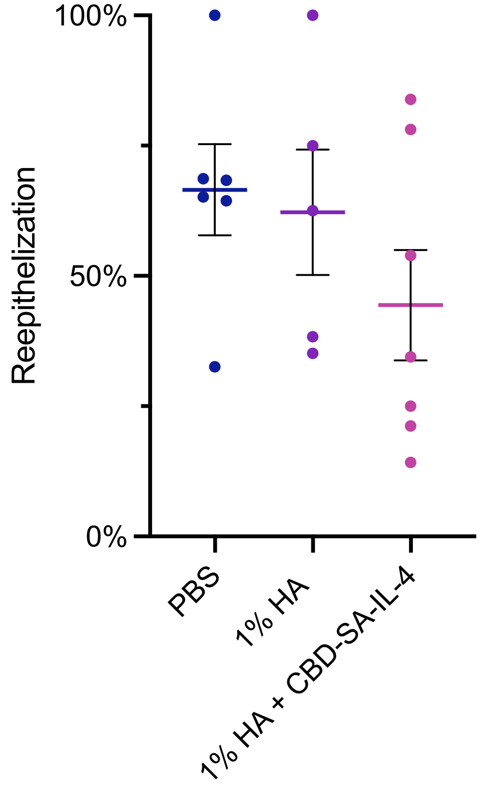

Supplementary Figure 3: Healthy (C57BL/6) reepithelization via H&E-stained quantification 5 days post wound creation (QuPath, See Methods) (PBS n=6, 1% HA n=5, 1% HA + CBD-SA-IL-4 n=6; mean ± SEM).

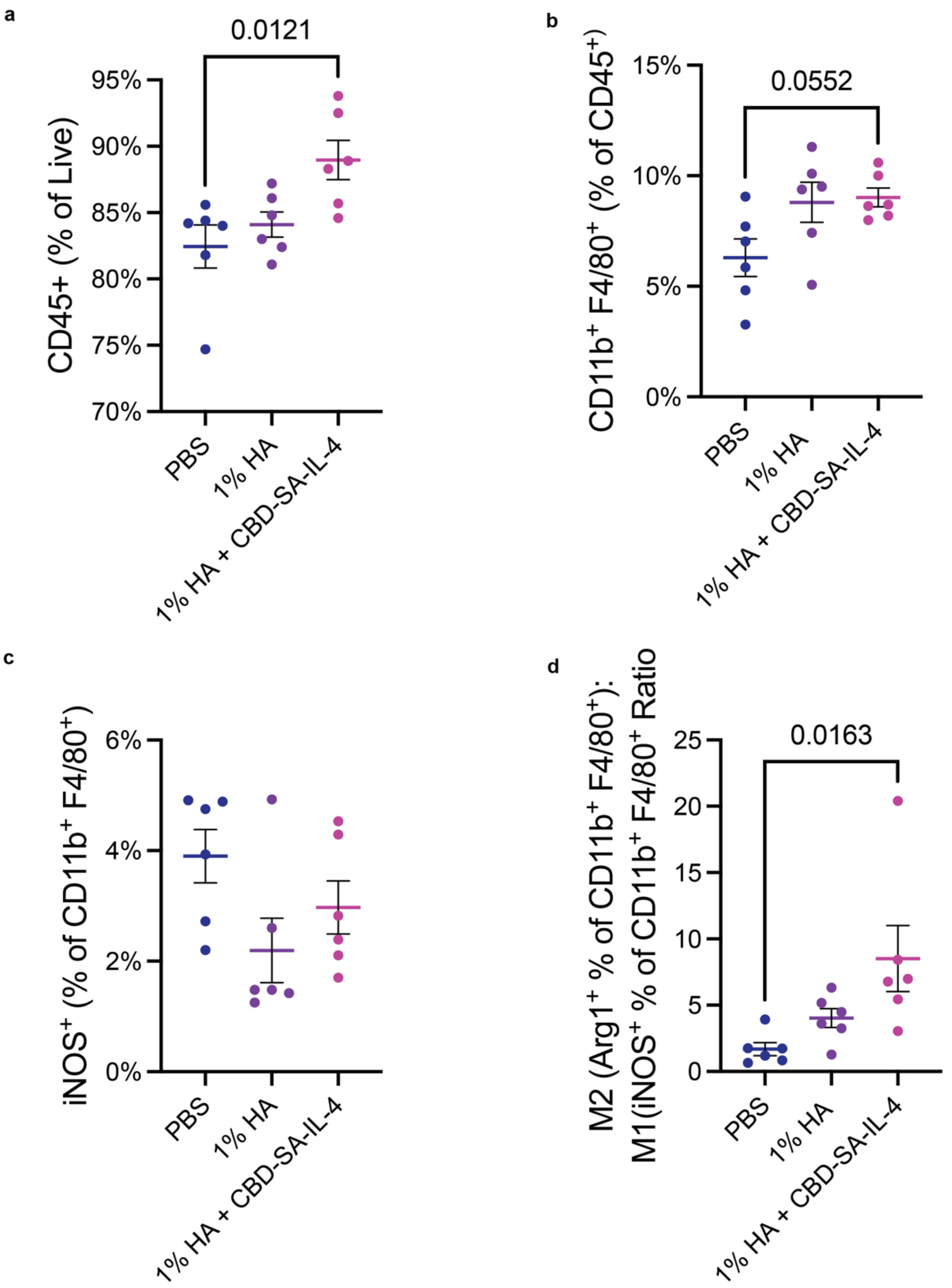

Supplementary Figure 4: CBD-SA-IL-4 increases immune cell infiltration to the wound, under the same experimental conditions as Figure 4. **a** CBD-SA-IL-4 treatment increases the immune cell ($CD45^+$) infiltration to the wound. **b** CBD-SA-IL-4 treatment shows trend towards increased macrophage compartment in the wound. **c** iNOS expression of wound macrophages, CBD-SA-IL-4 trends toward less expression. **d** Ratio between M2 and M1 macrophages is significantly increased following CBD-SA-IL-4 treatment. Statistics: **a-d** Analyzed using ordinary on-way ANOVA with Tukey multiple comparison of each group against all other groups; * $p<0.05$, ** $p<0.01$, *** $p<0.001$.

Supplementary Table 1: Amino Acid Sequence of CBD-SA-IL-4

CBD-SA-IL-4 Protein Sequence	METDTLLLWVLLLWVPGSTGDGPDCSQPLDVILLLDGSSSFPASYFDEMKSFAKAFISK ANIGPRLTQVSVLQYGSITTIDVPWNVVPEKAHLLSLVDVMQREGGPSQIGDALGFAVR YLTSEMHGARPGASKAVVILVTDVSVDSVDAAADAARSNRVTVFPIGIGDRYDAAQLRIL AGPAGDSNVVKLQRIEDLPTMVTLGNSFLHKLCSGFVRI<br>GGGSGGGSEAHKSEIAHRYNDLGEQHFKGLVLIAFSQYLQKCSYDEHAKLVQEVTDFA KTCVADESAANCDKSLHTLFGDKLCAIPNLRENYGELADCCTKQEPERNECFLQHKDD NPSLPPFERPEAEAMCTSFKENPTTFMGHYLHEVARRHPYFYAPELLYYAEQYNEILTQ CCAEADKESCLTPKLDGVKEKALVSSVRQRMKCSSMQKFGERAFKAWAVARLSQTFP NADFAEITKLATDLTKVNKECCHGDLLECADDRAELAKYMCENQATISSKLQTCCDKPL LKKAHCLSEVEHDTMPADLPAIAADFVEDQEVCKNYAEAKDVFLGTFLYEYSRRHPDYS VSLLLRLAKKYEATLEKCCAEANPPACYGTVLAEFQPLVEEPKNLVKTNCDLYEKLGEY GFQNAILVRYTQKAPQVSTPTLVEAARNLGRVGTKCCTLPEDQRLPCVEDYLSAILNRV CLLHEKTPVSEHVTKCCSGSLVERRPCFSALTVDETYVPKEFKAETFTFHSDICTLPEKE KQIKKQTALAELVKHKPKATAEQLKTVMDDFAQFLDTCCKAADKDTCFSTEGPNLVTRC KDALAGGGGSGGGSMHIHGCDKNHLREIIGILNEVTGEGTPCTEMDVPNVLTATKNTTE SELVCRASKVLRIFYLKHGKTPCLKKNSSVLMELQRLFRAFRCLDSSISCTMNESKSTSL KDFLESLKSIMQMDYSHHHHHH

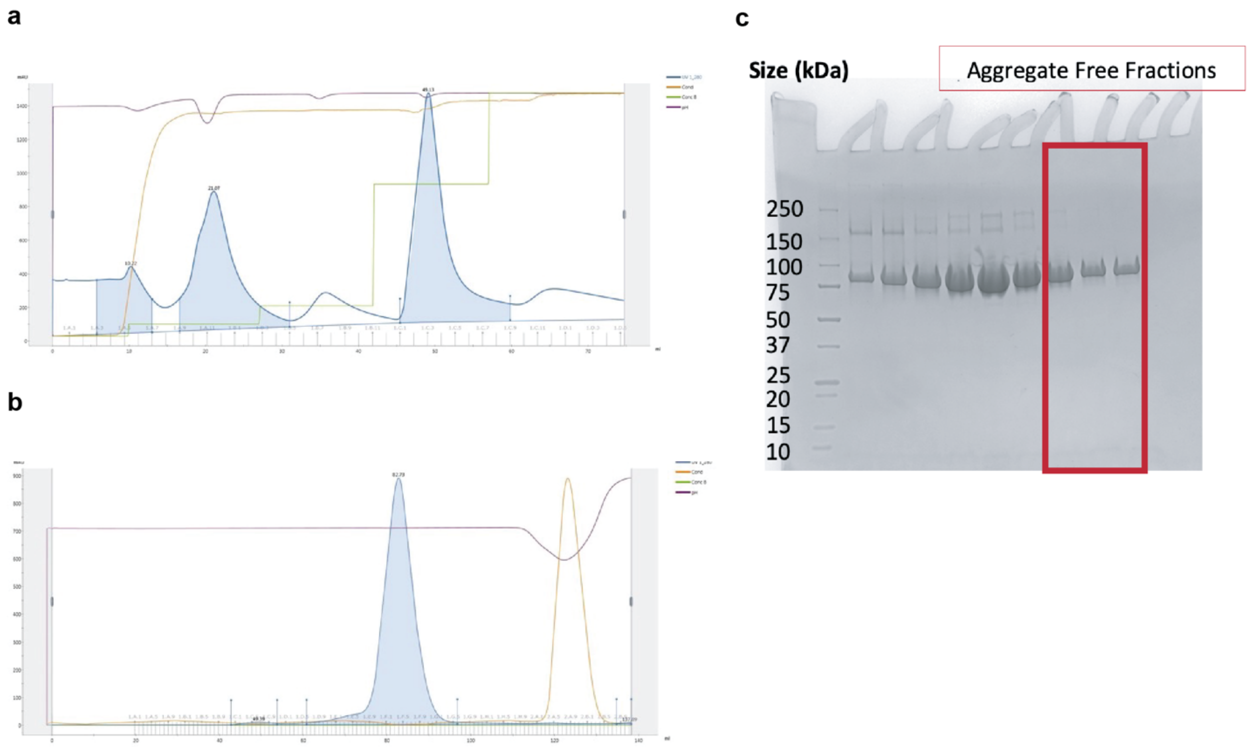

Supplementary Figure 5: Purification of CBD-SA-IL-4. **a** His purification trace showing elution of CBD-SA-IL-4 50% elution buffer. **b** Size exclusion column trace demonstrating elution of CBD-SA-IL-4 after approximately 70 mL of volume, corresponding to its size. **c** SDS-page showing CBD-SA-IL-4 after His purification and subsequent size exclusion column purification. Boxed portion depicts fractions collected for use.

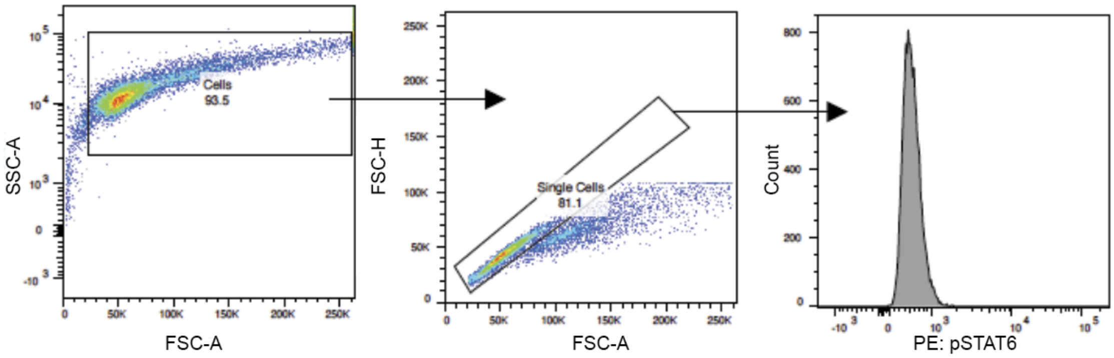

Supplementary Figure 6: Gating strategy for phosphorylated STAT6 assay using RAW264.7 macrophage cell line.

a

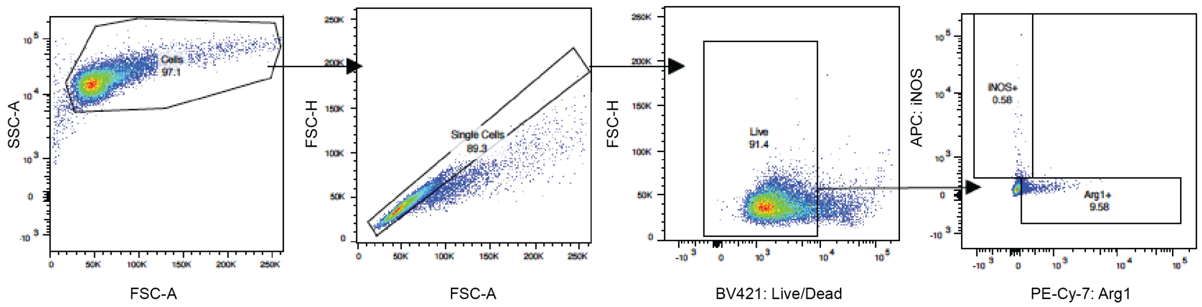

b

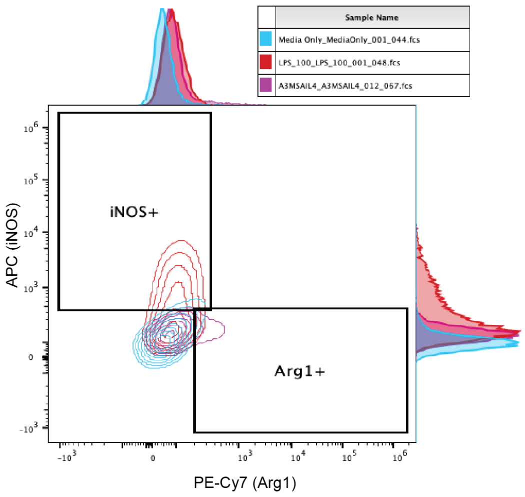

Supplementary Figure 7: Gating strategy for macrophage polarization assay using RAW264.7 macrophage cell line. **a** Gating strategy for macrophage polarization assay. **b** Representative plot and histogram for polarization state for media only (blue), LPS (red), and CBD-SA-IL-4 (pink) treated RAW264.7 cells.

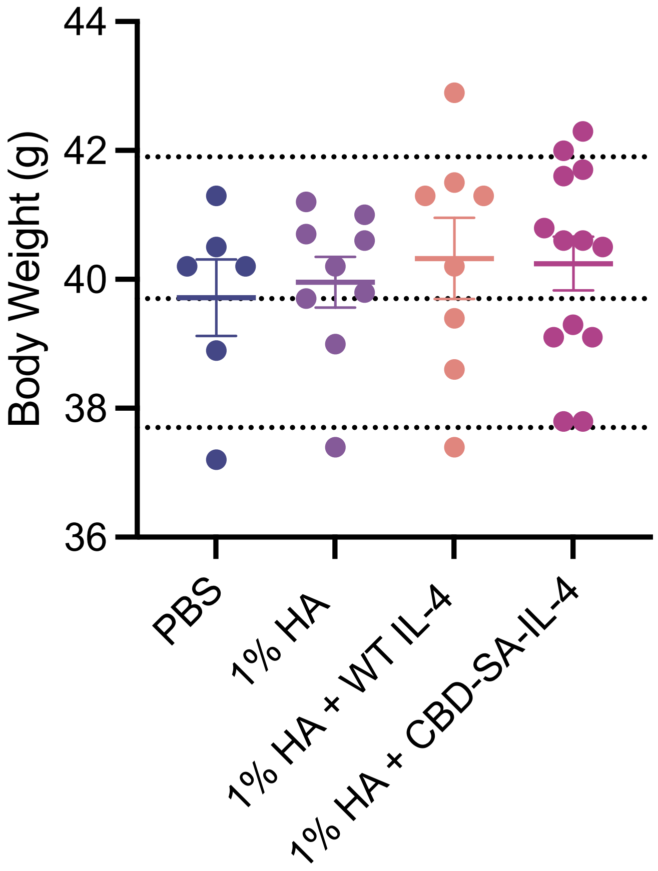

Supplementary Figure 8: Body weight of male db/db mice between ages 8 weeks – 10 weeks, taken pre-operatively. (PBS n=6, 1% HA n=8, 1% HA + WT IL-4 n=8, 1% HA + CBD-SA-IL-4 n=13, mean ± SEM)

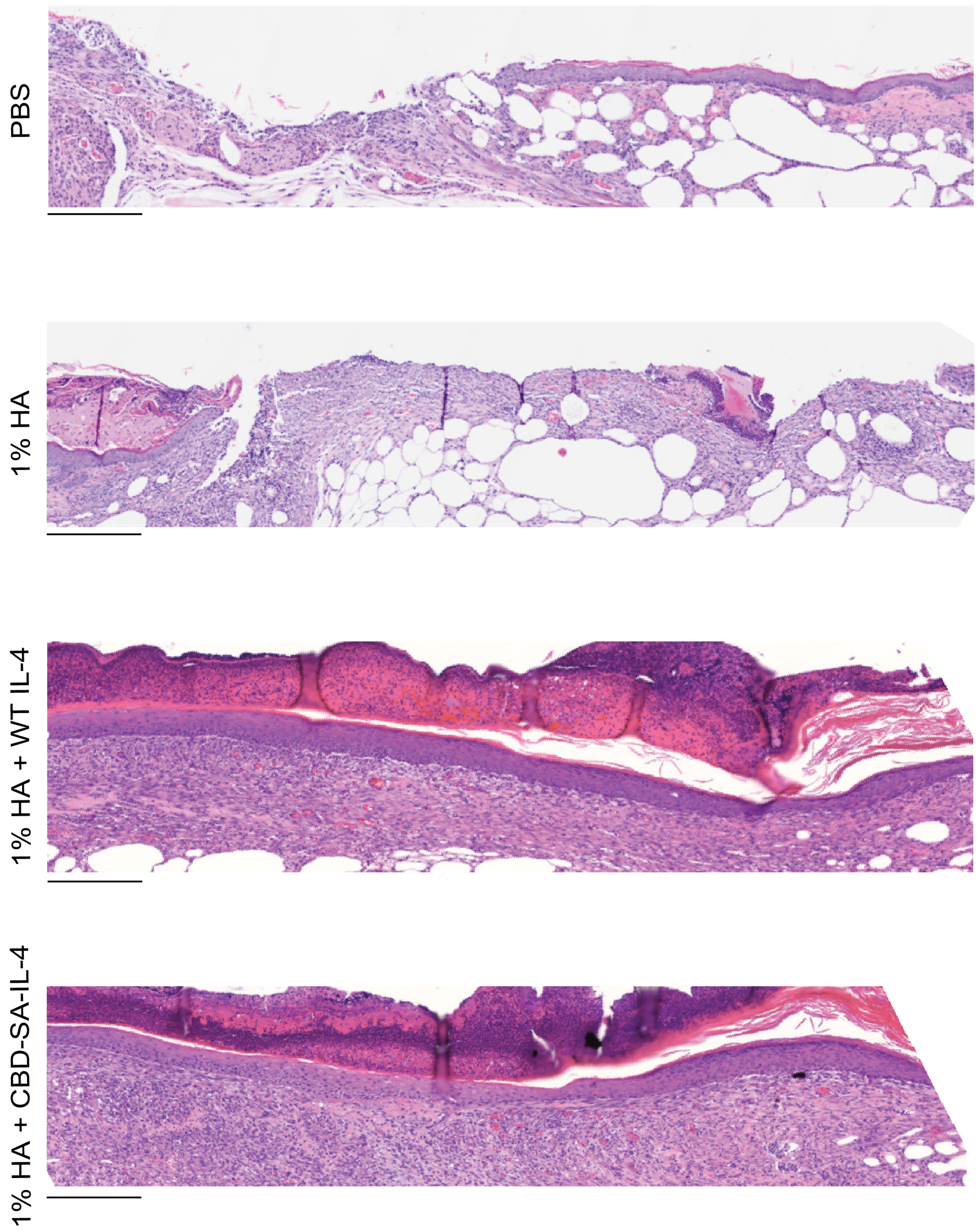

Supplementary Figure 9: Enlarged representative H&E images to better show epithelium.

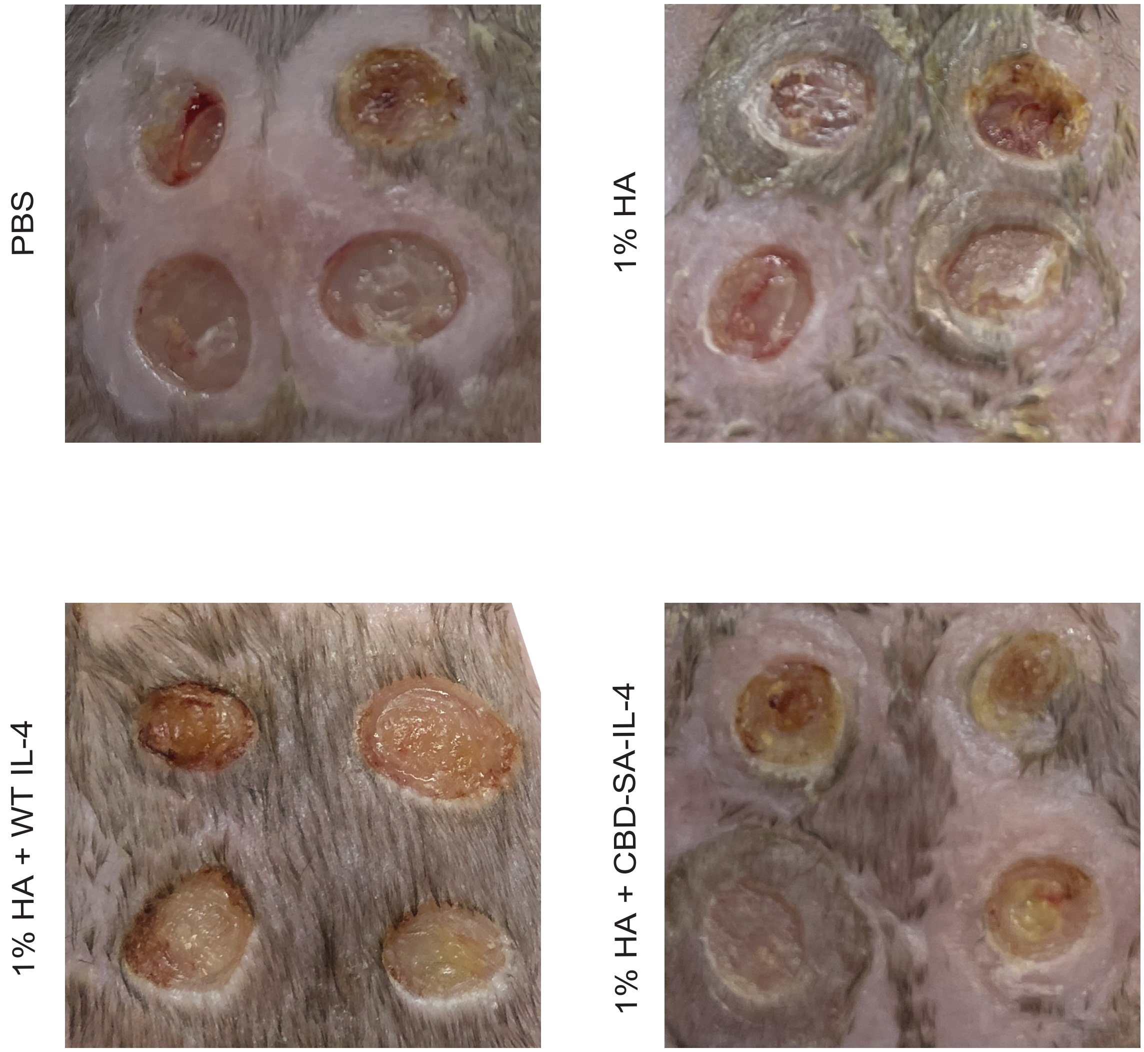

Supplementary Figure 10: Photos of day 11 wounds prior to excisional removal.

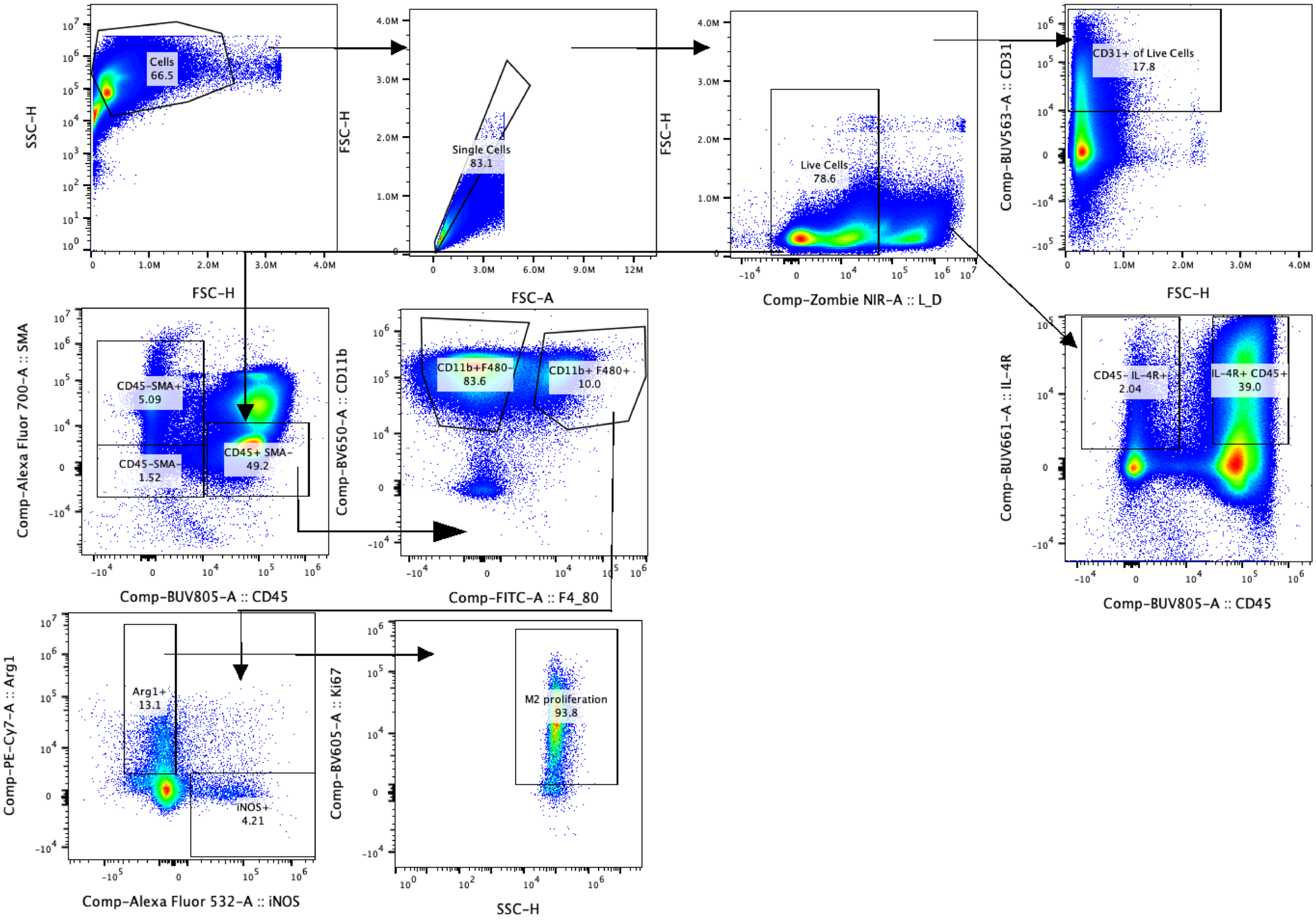

Supplementary Figure 11: Gating strategy for excised wound four days post treatment.
