# Supplementary material for: Topically-applied collagen-binding serum albumin-fused interleukin-4 modulates wound microenvironment in non-healing wounds
Source: NPJ Regen Med. 2023 Sep 11;8:49. doi: 10.1038/s41536-023-00326-y (PMC10495343; doi:10.1038/s41536-023-00326-y)
Supplement: Supplementary file 2 — Reporting Summary [file 41536_2023_326_MOESM2_ESM.pdf]

nature portfolio

Corresponding author(s): Jeffrey A. Hubbell & Jun Ishihara

Last updated by author(s): Mar 26, 2023

Reporting Summary

Nature Portfolio wishes to improve the reproducibility of the work that we publish. This form provides structure for consistency and transparency in reporting. For further information on Nature Portfolio policies, see our Editorial Policies and the Editorial Policy Checklist.

Statistics

For all statistical analyses, confirm that the following items are present in the figure legend, table legend, main text, or Methods section.

n/a	Confirmed	
☐	☒	The exact sample size (*n*) for each experimental group/condition, given as a discrete number and unit of measurement
☐	☒	A statement on whether measurements were taken from distinct samples or whether the same sample was measured repeatedly
☐	☒	The statistical test(s) used AND whether they are one- or two-sided<br>*Only common tests should be described solely by name; describe more complex techniques in the Methods section.*
☒	☐	A description of all covariates tested
☐	☒	A description of any assumptions or corrections, such as tests of normality and adjustment for multiple comparisons
☐	☒	A full description of the statistical parameters including central tendency (e.g. means) or other basic estimates (e.g. regression coefficient) AND variation (e.g. standard deviation) or associated estimates of uncertainty (e.g. confidence intervals)
☐	☒	For null hypothesis testing, the test statistic (e.g. *F*, *t*, *r*) with confidence intervals, effect sizes, degrees of freedom and *P* value noted<br>*Give P values as exact values whenever suitable.*
☒	☐	For Bayesian analysis, information on the choice of priors and Markov chain Monte Carlo settings
☒	☐	For hierarchical and complex designs, identification of the appropriate level for tests and full reporting of outcomes
☒	☐	Estimates of effect sizes (e.g. Cohen's *d*, Pearson's *r*), indicating how they were calculated

Our web collection on statistics for biologists contains articles on many of the points above.

Software and code

Policy information about availability of computer code

Data collection	Image Lab™ Software (v 5.2.1) (BioRAD), BD FACSDIVA™ SOFTWARE v8.0.1
Data analysis	Graph Pad Prism (v9.3.1) and Graph Pad online tools were used for analysis, FlowJo (v10.8.1), Image Lab™ Software (v 5.2.1) (BioRAD), Microsoft Excel for Mac v16.64,

For manuscripts utilizing custom algorithms or software that are central to the research but not yet described in published literature, software must be made available to editors and reviewers. We strongly encourage code deposition in a community repository (e.g. GitHub). See the Nature Portfolio guidelines for submitting code & software for further information.

Data

Policy information about availability of data

All manuscripts must include a data availability statement. This statement should provide the following information, where applicable:

- Accession codes, unique identifiers, or web links for publicly available datasets
- A description of any restrictions on data availability
- For clinical datasets or third party data, please ensure that the statement adheres to our policy

The authors declare that all data supporting the results in this study are available within the paper and its Supplementary Information. The datasets generated and analysed during the study are available from the corresponding author upon reasonable request.

Human research participants

Policy information about studies involving human research participants and Sex and Gender in Research.

Reporting on sex and gender	*Use the terms sex (biological attribute) and gender (shaped by social and cultural circumstances) carefully in order to avoid confusing both terms. Indicate if findings apply to only one sex or gender; describe whether sex and gender were considered in study design whether sex and/or gender was determined based on self-reporting or assigned and methods used. Provide in the source data disaggregated sex and gender data where this information has been collected, and consent has been obtained for sharing of individual-level data; provide overall numbers in this Reporting Summary. Please state if this information has not been collected. Report sex- and gender-based analyses where performed, justify reasons for lack of sex- and gender-based analysis.*
Population characteristics	*Describe the covariate-relevant population characteristics of the human research participants (e.g. age, genotypic information, past and current diagnosis and treatment categories). If you filled out the behavioural & social sciences study design questions and have nothing to add here, write "See above."*
Recruitment	*Describe how participants were recruited. Outline any potential self-selection bias or other biases that may be present and how these are likely to impact results.*
Ethics oversight	*Identify the organization(s) that approved the study protocol.*

Note that full information on the approval of the study protocol must also be provided in the manuscript.

Field-specific reporting

Please select the one below that is the best fit for your research. If you are not sure, read the appropriate sections before making your selection.

☒ Life sciences ☐ Behavioural & social sciences ☐ Ecological, evolutionary & environmental sciences

For a reference copy of the document with all sections, see nature.com/documents/nr-reporting-summary-flat.pdf

Life sciences study design

All studies must disclose on these points even when the disclosure is negative.

Sample size	Statistical methods were not used to predetermine the necessary sample size, rather sample sizes were chosen based on estimates from pilot experiments and previously published results such that appropriate statistical tests could yield significant results.
Data exclusions	Pre-determined exclusion criteria was established for wound healing efficacy study while blinded and was as follows: 1. If the splint was no longer adhered to the wound then the wound was excluded. 2. If upon review of histology slides the wound looked overtly damaged (no wound margin visible, breaks were seen in tissue) the sample was excluded.
Replication	Efficacy data was replicated three times, all other data was not repeated unless otherwise stated.
Randomization	Mice were purchased form vendors, numbered, and then mice were selected at random.
Blinding	The investigators were blinded to group allocation during data collection and analysis

Reporting for specific materials, systems and methods

We require information from authors about some types of materials, experimental systems and methods used in many studies. Here, indicate whether each material, system or method listed is relevant to your study. If you are not sure if a list item applies to your research, read the appropriate section before selecting a response.

Materials & experimental systems

n/a	Involved in the study	
☐	☒	Antibodies
☐	☒	Eukaryotic cell lines
☒	☐	Palaeontology and archaeology
☐	☒	Animals and other organisms
☒	☐	Clinical data
☒	☐	Dual use research of concern

Methods

n/a	Involved in the study	
☒	☐	ChIP-seq
☐	☒	Flow cytometry
☒	☐	MRI-based neuroimaging

Antibodies

Antibodies used	1. BD Horizon 564199 CD86 GL1 BUV395<br>2. BD OpiBuild 741251 CD31 MEC13.3 BUV563<br>3. BD OptiBuild 741557 CD124 mIL4R-M1 BUV661<br>4. BD OptiBuild 75215 CD45 13/2.3 BUV805<br>5. Biolegend 141717 CD206 C068C2 BV421<br>6. BD Horizon 563402 CD11b M1//70 BV650<br>7. Biolegend 117349 CD11c N418 BV711<br>8. Biolegend 123108 F4/80 BM8 FITC<br>9. BD Pharmagien 560602 Ly-6G IA8 PerCP-Cy5.5<br>10. Biolegend 107614 IA/IE (MHCII) M51114.15.2 APC<br>11. Biolegend 128045 Ly-6C HK1.4 APC-Fire750<br>12. Novus Biosciences MBP2-34522AF700 SMA 1A4/asm-1 AF700<br>13.Biolegend 652413 Ki67 16A8 BV605<br>14. Invitrogen 58-5920-80 iNOS CXNFT AF532<br>15.Invitrogen 25-3697-82 Arg1 AlexF5 PE-Cy7<br>16. Biolegend 423105 Live Dead Zombie NIR<br>17. BD Biosciences 562078 pSTAT-6 pY641<br>18. Invitrogen L34955 Fixable Violet Dead Cell (Live/Dead) BV421
Validation	All antibodies were purchased from the vendors mentioned above. These antibodies are routinely used in our laboratory without additional validation. Titration was preformed to validate panel design with specified antibodies

Eukaryotic cell lines

Policy information about cell lines and Sex and Gender in Research

Cell line source(s)	HEK-Blue™ TLR4 were purchased from Invitrogen. RAW264.7 cells were purchased from ATCC
Authentication	HEK-Blue™ TLR4 cells were treated with LPS, and found to activate in response to the TLR4 ligand. RAW264.7 cells were authenticated by vendor.
Mycoplasma contamination	Cells were tested for mycoplasma contamination, and all cell lines tested negative.
Commonly misidentified lines<br>(See ICLAC register)	No commonly misidentified cell lines were used.

Animals and other research organisms

Policy information about studies involving animals; ARRIVE guidelines recommended for reporting animal research, and Sex and Gender in Research

Laboratory animals	Unless otherwise specified mice were maintained in a specific pathogen-free facility at the University of Chicago. 8 to 10 week-old male BKS.Cg-Dock7m +/+ Leprdb/J (db/db) mice were purchased from The Jackson Laboratory. 8 to 10 week old C57BL/6 mice were purchased from The Jackson Laboratory and used as wild type mice in all studies unless otherwise indicated.
Wild animals	The study did not involve wild animals.
Reporting on sex	All mouse experiments were done using a single sex of mice. Male db/db were used due to the enhanced impairment in healing seen in males. Otherwise, female WT mice were used to enable co-housing of groups without inflammation due to aggression. Therefore sex-based analysis was not performed.
Field-collected samples	This study did not involve field collected samples.
Ethics oversight	All mouse studies were carried out in accordance with procedures approved by the Institutional Animal Care and Use Committee at the University of Chicago according to ACUP 72450.

Note that full information on the approval of the study protocol must also be provided in the manuscript.

Flow Cytometry

Plots

Confirm that:

☒ The axis labels state the marker and fluorochrome used (e.g. CD4-FITC).

☒ The axis scales are clearly visible. Include numbers along axes only for bottom left plot of group (a 'group' is an analysis of identical markers).

☒ All plots are contour plots with outliers or pseudocolor plots.

☒ A numerical value for number of cells or percentage (with statistics) is provided.

Methodology

Sample preparation	Wounded skin was removed with 6 mm biopsy punch and cut into small pieces (<5 mm2) and transferred to 4 mL of an enzyme solution (Liberase TL (0.4 mg/mL) Roche, DNase I (13.7 pU/mL) MP Biomedicals)), and incubated for 2 hr at 37°C. Then, the cells from the digested wounds were resuspended in 25 mL of media, passed through a 70 µm cell strainer, and centrifuged. The single cell suspension was counted, and 2x106 cells/mL were plated and stained for 15 min in 50 µL live/dead fixable dye and anti CD16/32 (BioLegend). After one wash. cells were stained for 30 min in 50 µL of 2% FBS in PBS-containing antibodies (Table 1). Intracellular staining was performed using the eBiosciene Foxp3/Transcription Factor Staining Buffer Set according to the manufacturer's instructions (Invitrogen). Cells were analyzed using an Aurora (Cytek) spectral flow cytometer and FlowJo software (FlowJo, LLC).
Instrument	Sample collection was done on a BD LSR flow cytometer or Cytek AURORA
Software	FlowJo (v10) was used to analyse flow cytometry data.
Cell population abundance	No cell sorting was used. Sample preparation described above.
Gating strategy	The gating strategy is outlined in the Supplementary Information.

☒ Tick this box to confirm that a figure exemplifying the gating strategy is provided in the Supplementary Information.
